# Hypertensive Patients Exhibit Enhanced Thrombospondin-1 Levels at High-Altitude

**DOI:** 10.3390/life11090893

**Published:** 2021-08-29

**Authors:** Kavita Sharma, Neha Chanana, Ghulam Mohammad, Tashi Thinlas, Mohit Gupta, Mansoor Ali Syed, Rajat Subhra Das, Qadar Pasha, Aastha Mishra

**Affiliations:** 1 Cardiovascular Respiratory Disease Unit, CSIR-Institute of Genomics and Integrative Biology, Delhi 110007, India; kavita.sharma@igib.in (K.S.); chanananeha25@gmail.com (N.C.); qpasha@igib.in (Q.P.); 2 Department of Biotechnology, Jamia Millia Islamia, New Delhi 110025, India; smansoor@jmi.ac.in; 3 Department of Medicine, SNM Hospital, Leh 194101, India; ghulamnmohd@yahoo.com (G.M.); tashithinlas@gmail.com (T.T.); 4 Department of Cardiology, GB Pant Institute of Post Graduate Medical Education and Research, New Delhi 110002, India; drmohitgupta@yahoo.com; 5 Department of Anatomy, All India Institute of Medical Sciences, Raebareli 229405, India; rajatsubhrad0@gmail.com; 6 Indian Council of Medical Research, New Delhi 110029, India; 7 Academy of Scientific and Innovative Research (AcSIR), Ghaziabad 201002, India

**Keywords:** thrombospondin-1, cluster of differentiation, hypertension, high-altitude, hypobaric hypoxia, gene variants

## Abstract

Thrombospondin-1 (THBS1) levels elevate under hypoxia and have relevance in several cardiovascular disorders. The association of THBS1 with endothelial dysfunction implies its important role in hypertension. To establish the hypothesis, we screened patients with hypertension and their respective controls from the two different environmental regions. Cohort 1 was composed of Ladakhis, residing at 3500 m above sea level (ASL), whereas Cohort 2 was composed of north-Indians residing at ~200 m ASL. Clinical parameters and circulating THBS1 levels were correlated in the case–control groups of the two populations. THBS1 levels were significantly elevated in hypertension patients of both cohorts; however, the levels were distinctly enhanced in the hypertensive patients of HA as compared to normoxia (*p* < 0.002). The observation was supported by the receiver operating curve analysis with an area under curve of 0.7007 (0.627–0.774) demonstrating the discriminatory effect of hypobaric hypoxia on the levels as compared to normoxia (*p* < 0.011). Significant correlation of THBS1 and mean arterial pressure was observed with upraised positive correlations in the hypertensive highlanders as compared to the hypertensive patients from sea-level. The prevalence of differential distribution of *THBS1* and *CD47* genes variants, their interactions, and association with the THBS1 levels were also determined. Genotype-interactions between *THBS1* rs2228263 and *CD47* rs9879947 were relevant and the regression analysis highlighted the association of risk genotype-interactions with increased THBS1 levels in hypertension. Genetic studies of additional thrombospondin pathway-related genes suggest the complex role of THBS1 in the presence of its family members and the related receptor molecules at HA.

## 1. Introduction

Thrombospondin-1 (THBS1), expressed in various cells like endothelial cells, vascular smooth muscle cells (VSMCs), and cardiomyocytes, elevates dramatically under conditions of hypoxia [1]. THBS1, in fact, belongs to the thrombospondin (THBS) family consisting of five multifunctional glycoproteins, namely THBS1–5. Out of these, THBS1 and 2 have been studied the most and are described in cell–cell interactions, apoptosis and as anti-angiogenic agents [2]. THBS1 functions mainly through its receptor CD47 [3]; however, the other receptors are also involved [4]. The functions of markedly induced levels of THBS1 are confirmed within the tumor microenvironment [5] and in cardiovascular disorders [3]. The endothelium, a critical regulator of vascular health and blood pressure, regulates VSMCs contractions and platelet aggregations [6]. THBS1 inhibits the endothelial functions through various mechanisms of action [7,8,9,10]. The binding of THBS1 with CD47 inhibits the soluble guanylyl cyclase and protein kinase G in VSMCs, disrupting the nitric oxide (NO) and vascular endothelial growth factor (VEGF) pathways [8]. THBS1 in higher concentrations binds to CD36 to decrease intracellular cyclic GMP levels and endothelial nitric oxide synthase-dependent synthesis of NO [9]. THBS1 also exerts its biological effects by activating transforming growth factor β (TGF-β), leading to excessive fibrosis [10]. Hence, THBS1 invariably associates with endothelial dysfunction by inducing the onset of inflammatory reactions and is involved in the process of platelet aggregation and adhesion to endothelial cells. All of these functions of THBS1 imply its important role in hypertension pathophysiology. Of note, a significant percentage of permanent residents of high-altitude (HA) suffer from hypertension [11]. Moreover, blood pressure elevates and persists for a longer period in sojourners visiting HA, leading to the hypertension status in them [11]. Such observations made us hypothesize the role of THBS1 and the other thrombospondin family members, both genes and proteins, in hypertension at HA. Additionally, the hypobaric hypoxia exposures at HA induce remodeling of pulmonary circulation, which is a complex process involving numerous interactive events [12]. The stimulation of THBS1 in hypoxia [1] and its role in modulation of vascular smooth muscle cell proliferation and migration [13], further makes it an important candidate molecule to be explored at HA.

To establish our hypothesis, we evaluated the THBS1 levels in the hypertension patients residing at HA. However, in the process, we also made a comparison with hypertension patients residing at sea-level of the Indian sub-continent. The present study, thus, screened two cohorts belonging to two different ethnicities. The first cohort of Tibeto-Burman descent consisted of Ladakhi, 3500 m above sea-level (ASL) hypertensive patients with their respective normotensive highland controls. In contrast, the second cohort of Indo-Aryan descent consisted of hypertensive patients at sea-level, ~200 m ASL with their normotensive controls. Apart from evaluating circulating THBS1 levels in the two cohorts, we also investigated the possible association of the THBS1 levels with clinical variables. Furthermore, we also determined the prevalence of differential distribution of gene variants in *THBS1* and *CD47*, the gene–gene interactions, and their association with the THBS1 levels in the two study cohorts. The identification and screening of additional thrombospondin pathway-related genes and their variants was performed in the HA cohort to uncover the molecular mechanism of enhanced THBS1 levels in the hypertensive patients residing at HA and their plausible genetic influences under this extreme condition. The study was worth undertaking because we could find enhanced levels of THBS1 in hypertension under the hypobaric hypoxic condition at HA; the levels being comparatively higher than their sea-level counterparts.

## 2. Materials and Methods

### 2.1. Study Participants and Clinical Evaluation

The study was approved by the Human ethics committees of Council of Scientific and Industrial Research-Institute of Genomics and Integrative Biology (CSIR-IGIB), Delhi; SNM Hospital, Leh, Ladakh, India and GB Pant Institute of Post Graduate Medical Education and Research, Delhi, India. In addition, informed and written consent was obtained from each participant.

In an ethnically matched case–control study, a total of 1388 subjects were recruited for genotyping from two different altitude regions of India and were categorized into two major study cohorts, namely, Ladakhi highlanders residing at altitude ≥ 3500 m (Cohort 1, *n* = 349) of Tibeto-Burman ethnicity and North Indian lowlanders living at the altitude of <200 m (Cohort 2; *n* = 1039) of Indo-Aryan ethnicity. Cohort 1 is composed of healthy normotensive highlanders (NHLs, *n* = 183) as controls and hypertensive highlanders (HHLs, *n* = 166) as the cases. Likewise, Cohort 2 is composed of healthy normotensive lowlanders (NLLs, *n* = 517) as controls and hypertensive lowlanders (HLLs, *n* = 522) as the patients.

Control recruitment criteria included age 25–60 years, systolic blood pressure (SBP) < 120 mmHg and diastolic blood pressure (DBP) < 80 mmHg, absence of a family history of hypertension, and any disease medication. Patient’s recruitment criteria were age 25–60 years, SBP ≥ 140 mmHg and/or DBP ≥ 90 mmHg (JNC VII), and absence of antihypertensive medication. All the subjects were rested for 5 min prior to blood pressure (BP) measurement. In a supine position, three measurements of BP using a calibrated mercury sphygmomanometer with appropriate adult cuff size were recorded by trained clinicians. The point at which the first of two or more Korotkoff sounds was heard was recorded as SBP and the disappearance of Korotkoff sound as DBP.

Participants of the study unwilling to consent or on medication were excluded from the study. Few subjects were also removed to maintain the matching age and the male to female ratio in the study. Moreover, physical examination and laboratory tests excluded individuals with coronary artery disease, vascular disease, stroke, secondary hypertension, diabetes mellitus, and renal diseases. Blood was drawn in a supine position after overnight fasting. Peripheral blood leukocytes were used for DNA extraction and plasma to analyze biochemical parameters; DNA samples were stored at −20 °C and plasma at −80 °C if not used immediately.

### 2.2. Thrombospondin-1 Circulating Levels Measurement

Human plasma THBS1 levels were determined using a biotin-avidin associated sandwich Enzyme-Linked Immunosorbent Assay Kit (USCN Life Science Inc., Wuhan, China). Estimation of THBS1 was performed in 83 subjects, each belonging to the NHLs and HHLs of Cohort 1 and 89 subjects each belonging to the NLLs and HLLs of Cohort 2. The intensity of the color developed was directly proportional to the concentration of THBS1 in the sample and was measured at the wavelength 450 nm on a high-throughput SpectraMaxplus384 Spectrophotometer (Molecular Devices, Sunnyvale, CA, USA). The inference was drawn from internal standards.

### 2.3. Receiver Operating Characteristic Curve

Receiver operator characteristics (ROC) analysis was performed to determine the influence and association of HA on THBS1 levels. The sea-level hypertensive group (HLLs) was compared with the hypertensive highlanders (HHLs). The sensitivity was plotted against 100-specificity and area under the curve (AUC) with 95% confidence interval (95% CI). It provided an index of association between HA and hypertension; a higher AUC reflects a greater association. A *p*-value ≤ 0.05 was considered as statistically significant.

### 2.4. Correlation Analysis

The plasma THBS1 levels and mean arterial pressure (MAP) were analyzed for correlation in the study cohorts using Statistical Package for Social Sciences version 16.0 (SPSS 16.0; SPSS Inc., Chicago, IL, USA) to ascertain any association between clinical and phenotypic findings.

### 2.5. Selection and Screening of the *THBS1* and *CD47* Gene Variants

Candidate genes *THBS1* and *CD47* were screened for possible polymorphisms based on their spanning the regulatory regions, i.e., promoter, 5′UTR and 3′UTR or presence in the exonic regions, i.e., missense synonymous SNPs were preferred over intronic SNPs and were selected based on the clinical and functional relevance. This extensive literature survey, including HapMap and Indian Consortium genomic data, provided us with *THBS1* SNP rs2228263 and *CD47* SNP rs9879947 associating with cardiovascular, pulmonary, and other diseases [14,15,16,17].

#### 2.5.1. SNP Genotyping in Highland and Sea-Level Populations

Genotyping of *THBS1* SNP rs2228263 and *CD47* SNP rs9879947 were performed by Fluidigm 48.48 SNPtype assay of the two customized SNPs in both study cohorts using the high-throughput Nanofluidic Dynamic Arrays from Fluidigm dynamic array integrated fluidic circuit (IFC) technique on a Biomark HD System (Fluidigm Corporation, San Francisco, California, CA, USA) as per manufacturer’s protocol. The genotype data for the two cohorts were analyzed using the BioMark SNP Genotyping Analysis software version 3.1.2. The software calculated the FAM, VIC, or HEX fluorescence intensities relative to ROX fluorescence background and then automatically classified the samples into three possible genotypes. Samples with a call rate below 90% were removed from the analysis. For all the genotyped SNPs in the two study cohorts, their role in hypertension was established by multivariate logistic regression analysis using SPSS 16.0.

#### 2.5.2. SNP–SNP Interactions and Allelic Influences of *THBS1* and *CD47* Gene Variants

Determining the association of polymorphisms with common, complex diseases can be difficult because any single SNP’s effect will likely be dependent on other SNPs, i.e., gene–gene interactions or epistasis. SNP–SNP interactions were performed using *THBS1* rs2228263 and *CD47* rs9879947 to reveal the best locus-model that deciphered allelic influences through the multifactor dimensionality reduction (MDR) method. MDR identified the best disease-predicting model based on interacted-genotypes carrying a different set of risk alleles.

### 2.6. Evaluation of THBS and CD Family Variants

The plasma THBS1 levels, ROC curve and differential *THBS1* and *CD47* gene variants in the study cohorts indicated the significance of the THBS1 gene family under hypobaric hypoxia. The interesting findings persuaded us to include the *THBS* and *CD* related genes and their SNPs for further screening in Cohort 1 (the Ladakhi highland population). We screened 122 SNPs belonging to 34 genes using Fluidigm 48.48 SNPtype assay. The high-throughput Nanofluidic Dynamic Arrays from Fluidigm dynamic array integrated the fluidic circuit (IFC) technique on a Biomark HD System (Fluidigm Corporation, San Francisco, CA, USA) and was used as per manufacturer’s protocol. Details of all the studied SNPs are provided in Table S1. Samples with a call rate below 90% were removed from the analysis. SNPs with a call rate of more than 95% and polymorphic were included for further analysis. The call rate cut-offs for samples and the SNPs were decided based on the available literature on Fluidigm SNP genotyping data analyses.

#### 2.6.1. Identifying Hypertension Associated Variants

We employed the PLINK v1.07 tool for the quality control (QC)-passed extensive genetic dataset analysis with 122 SNPs. The association with hypertension was deduced by logistic regression analysis under a log-additive model adjusting for age and gender. It provided the top significant SNPs.

#### 2.6.2. Multi-Locus Interactions for Association Analysis

The combined effect of the 122 SNPs located at a different locus, haplotype and SNP–SNP interaction analyses were performed in the study Cohort 1 to identify the multi-locus interactions of the genetic variants in various complex disease pathways. The pair-wise combinations of SNPs were tested using PLINK v1.07 to determine the epistasis. Haplotypes were generated, and the associations were deduced with general linear models (GLMs). For the disease traits, an omnibus association statistic was computed with linear and logistic regression analysis. This analysis provided us with the significant SNP–SNP interactions within the same and different gene(s).

### 2.7. Protein–Protein Interactions Network

According to Graph theory, the topological structure of the protein–protein interactions (PPI) network provides basic and direct information regarding the network of proteins and their associated biological functions. Combining the topological structure of the PPI network with the relevant biological knowledge provides a promising tool for understanding the biological mechanisms of species. Therefore, the significant genes related to the THBS and CD family, including *THBS1* and *CD47* were subject to the construction of PPI network by utilizing the STRING v11.0 database [18]. Available online: https://string-db.org (accessed on 24 June 2021). The gene ontology (GO) term and pathway analyses of our hypertension-associated genes in HA Cohort 1 were performed to provide us with GO-Cellular Component (CC), GO-Molecular Function (MF), GO-Biological Process (BP), and Reactome pathway collection, respectively. Each protein–protein interaction is annotated with one or more ‘scores’ that are indicators of confidence, i.e., how likely STRING judges an interaction true, given the available evidence. All scores rank from 0 to 1, with 1 being the highest possible confidence. For example, a score of 0.5 indicates that roughly every second interaction is erroneous (i.e., a false positive).

### 2.8. Statistical Analysis

The individual role of SNPs in hypertension and health was established by multivariate logistic regression analysis using SPSS 16.0 software. Genotype and allele distributions, OR and 95% CI were calculated, and *p*-values were adjusted with age and gender; significance was maintained at *p*-value ≤ 0.05 after false discovery rate (FDR) correction (BenjaminiHochberg.xlsx calculator). The significance of the screened SNPs was considered based on the different genetic models, i.e., co-dominant, recessive and allelic testing. Hardy–Weinberg equilibrium was checked using a χ^2^ goodness-of-fit test. Unpaired Student’s *t*-test was used to compare the differences in demographic features between the two groups, i.e., patients and controls in each cohort using EpiInfoTM v. 6 (Centre for Disease Control, Atlanta, GA, USA). THBS1 levels were log-transformed before analysis because of their skewed distribution and are expressed as mean ± SE (standard error), whereas the significance levels were analyzed with the general linear model (GLM) using SPSS 16.0. Pearson’s correlation (r) evaluated the correlation between the two continuous variables. The coefficient value between ±0.50 and ±1 is a strong correlation. The value between ±0.30 and ±0.49 is a medium correlation, while the value below ±0.29 is a weaker correlation. The combinations of genotypes were investigated for association with THBS1 levels. Correlation of interactions with levels was performed using ANCOVA (analysis of covariance) after adjustment with the confounding factors as mentioned above. Where appropriate, *p*-values for pair-wise differences were corrected for multiple comparisons by using Bonferroni correction and a *p*-value ≤ 0.05 was considered statistically significant.

## 3. Results

### 3.1. Evaluation of Demographic and Clinical Characteristics

Table 1 represents the demographic and clinical characteristics of the two study cohorts. The study cohorts comprised highlanders Cohort 1 of Tibeto-Burman ethnicity and North Indian lowlanders Cohort 2 of Indo-Aryan ethnicity. Both the cohorts included healthy controls and hypertensive patients. In Cohort 1, the age and body mass index (BMI) did not differ significantly between the patients and controls (*p* > 0.05) while they were significant in Cohort 2 study groups (*p* < 0.05). The SBP and DBP, and as a consequence, MAP was significantly elevated in patients as compared to the controls of both the cohorts (*p* < 0.0001). Age differed between the NLLs and HLLs. All the subsequent analyses used age and BMI as confounders.

### 3.2. Plasma THBS1 Levels Upregulate in Hypertensive Study Groups

**Figure 1:**
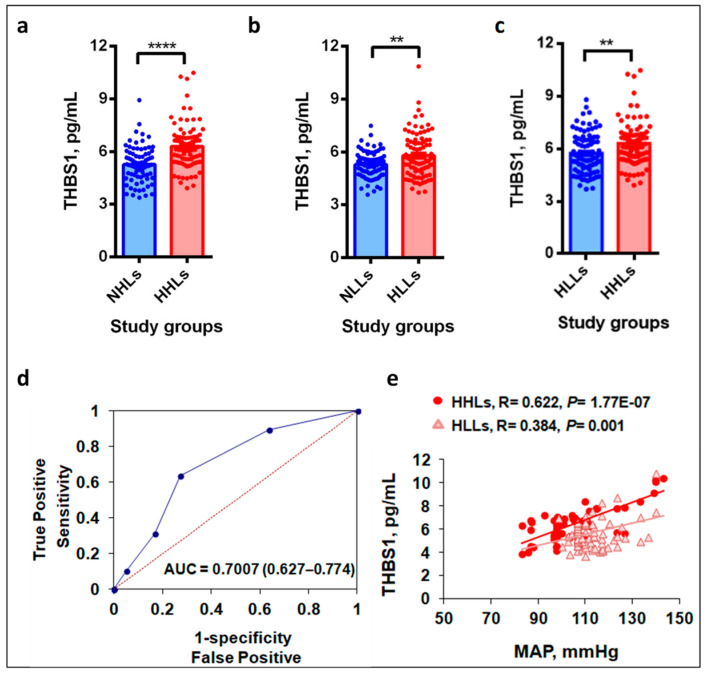
THBS1 levels under hypobaric hypoxia and normoxia. (**a**) Plasma levels of THBS1, pg/mL in Cohort 1, residing at HA. The levels were higher in the HHLs as compared to NHLs (*p* = 4.19 × 10^−8^), (**b**) plasma levels of THBS1, pg/mL in Cohort 2, residing at sea-level. The levels were higher in the HLLs as compared to NLLs (*p* < 0.002), (**c**) plasma levels of THBS1, pg/mL in hypertensive patients at high-altitude (HHLs) and at sea-level (HLLs). The levels were significantly higher in the HHLs as compared to HLLs (*p* = 0.002), (**d**) ROC curve exhibiting the significant AUC of 0.701 (0.627–0.774) to demonstrate the discriminatory effect of hypobaric hypoxia (HHLs) vs. normoxia (HLLs) on THBS1 levels in hypertension (*p* = 0.011), (**e**) scatter plot depicting correlation between THBS1 levels, pg/mL and MAP, mmHg under normoxia and hypoxia in Cohort 1 (*p* < 0.001). HHLs (hypoxia) showed more significance and positive correlation as compared to the HLLs (normoxia). Error bar represents standard error of the mean (SEM); statistical analysis was performed using Student’s *t*-test. Significance was maintained at *p* ≤ 0.05. THBS1, Thrombospondin 1; NHLs, normotensive highlanders; HHLs, hypertensive highlanders; NLLs, normotensive lowlanders; HLLs, hypertensive lowlanders; ROC, receiver operating characteristic curve; AUC, area under curve; MAP, mean arterial pressure. **** represents *p* values <0.0001 and ** represents *p* values <0.01.

Figure 1a,b exhibits significantly elevated plasma THBS1 levels in hypertensive patients, HHLs and HLLs from both cohorts compared to their respective controls, NHLs and NLLs. In Cohort 1, the levels were 6.30 ± 1.30 pg/mL in HHLs as compared to 5.26 ± 0.99 pg/mL in NHLs (*p* = 4.19 × 10^−8^; Figure 1a). Likewise, in Cohort 2, the THBS1 levels were 5.73 ± 1.15 pg/mL in HLLs as compared to 5.28 ± 0.72 pg/mL in NLLs (*p* = 0.002; Figure 1b). Although Cohort 2 was smaller in size, we still could observe the significantly enhanced THBS1 levels of 6.30 ± 1.30 pg/mL in hypertensive highlanders (HHLs) compared to the levels of 5.73 ± 1.15 pg/mL in the hypertensive patients from sea-level (HLLs, *p* = 0.002, Figure 1c).

### 3.3. Hypobaric Hypoxic Environment Enhances THBS1 Levels

The influence and association of HA on THBS1 levels were determined using ROC analysis that provided overall good discrimination based on the obtained results with AUC of more than 0.6 for THBS1 levels. Figure 1d exhibits a significant AUC (95% CI) of 0.7007 (0.627–0.774) that could differentiate hypertension at HA and sea-level in terms of THBS1 levels (*p* < 0.011).

### 3.4. THBS1 Levels Correlate with MAP

Figure 1e demonstrates a significant positive correlation between THBS1 levels and MAP in the patient groups, HHLs and HLLs of both cohorts (*p* < 0.001). Interestingly, Pearson’s correlation between THBS1 levels and MAP was stronger and more significant in the HHLs (R = 0.622, *p* = 1.77 × 10^−7^) compared to the HLLs (R = 0.384, *p* = 0.001). No correlation was found in the control groups; hence, the data is not shown.

### 3.5. Genotype Distributions of THBS1 rs2228263 and Its Receptor CD47 rs9879947

Multivariate logistic regression analysis after adjustment with age, gender and BMI revealed a higher risk towards hypertension for the prevalent *CD47* rs9879947AA homozygotes in HA hypertensive population (Table 2). The depicted results in Table 2 are based on the three genotype models i.e., co-dominant, recessive and allelic. On comparative genetic data analysis between the NHLs and HHLs, the prevalence of *CD47* rs9879947AA genotype in HHLs was significant as compared to rs9879947GG (*p* < 0.01). Consequently, the allele A was over-represented in HHLs and hence was recognized as a risk for hypertension with an OR (95% CI) of 2.56 (1.19–5.51). Likewise, the comparative genetic association analysis between the NLLs and HLLs identified *THBS1* rs2228263CC as the significantly associated protective genotype over-represented in NLLs compared to rs2228263TT with an OR (95% CI) of 0.40 (0.18–0.89; *p* = 0.024). At allelic level, however, the significance was lost (*p* > 0.05). The *CD47* rs9879947AA genotype was overrepresented in HLLs as compared to NLLs but was not statistically significant (*p* > 0.05).

#### 3.5.1. Effect of Risk Allele on the Circulating Levels

The linear regression exercise checked the causal effect of the *THBS1* risk allele with inconclusive observation. Clearly, a single allele change did not have a significant effect on the THBS1 levels. The genetic interactions between *THBS1* and *CD47* SNPs were explored in our further analysis that provided encouraging findings.

#### 3.5.2. Genetic Interactions between the *THBS1* and *CD47* SNPs Revealed the Causal Role of Risk Alleles

**Figure 2:**
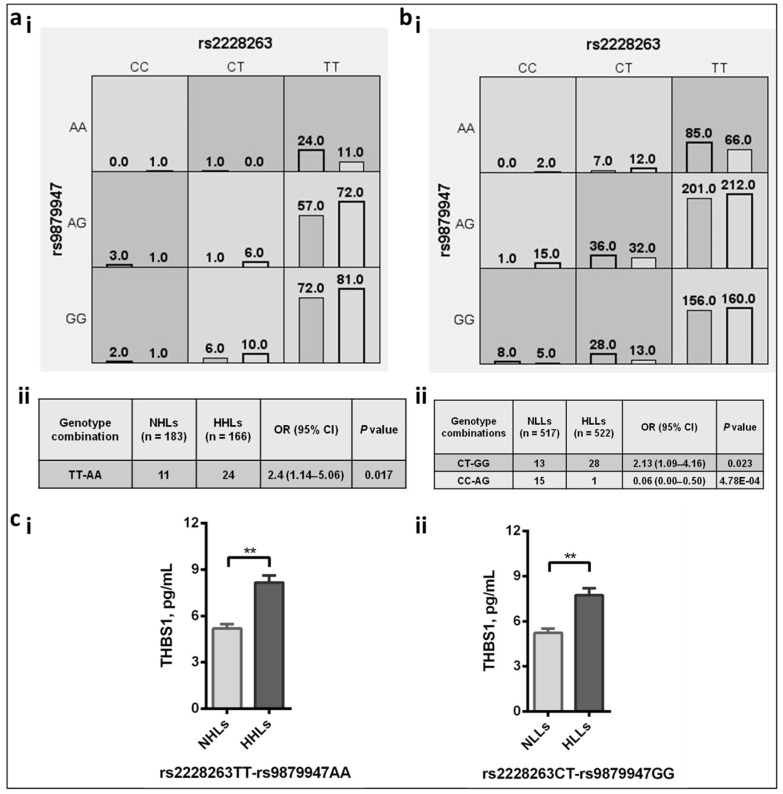
Multi-dimensional reduction results depicting genotype interactions between THBS1 and CD47 in high-altitude (Cohort 1) and sea-level (Cohort 2) populations. (**ai**) Distribution of genotype interactions between THBS1 rs2228263 and CD47 rs9879947 in a two-locus MDR model for Cohort 1. (**aii**) Significant genotype combination between NHLs and HHLs as observed between the THBS1 rs2228263 and CD47 rs9879947 in a two-locus model, (**bi**) distribution of genotype interactions between THBS1 rs2228263 and CD47 rs9879947 in a two-locus model for Cohort 2. (**bii**) Significant genotype combination between NLLs and HLLs as observed between the THBS1 rs2228263 and CD47 rs9879947 in a two-locus model, boxes with dark shade depict high risk, boxes with light shade depict low risk, and boxes with no shade/white depict blank; the latter represents no combination, (**ci**) correlation of the risk genotype combination rs2228263TT and rs9879947AA with the human plasma levels of THBS1, pg/mL in NHLs and HHLs (Cohort 1), (**cii**) correlation of the risk genotype combination rs2228263CT and rs9879947GG with the human plasma levels of THBS1, pg/mL in NLLs and HLLs (Cohort 2). Error bar represents standard error of the mean (SEM); the statistical analysis was done using Student’s *t*-test. THBS1, thrombospondin 1; NHLs, normotensive highlanders; HHLs, hypertensive highlanders; NLLs, normotensive lowlanders; HLLs, hypertensive lowlanders. ** represents *p* values less than <0.01.

In the two study cohorts, MDR analysis gave the two-locus model involving *THBS1* rs2228263 and *CD47* rs9879947 as the best model with consistency score 10/10 and the significant training and testing balanced accuracy (*p* < 0.05). Figure 2(ai,aii) compares the genotype data between NHLs and HHLs. The MDR derived rs2228263TT-rs9879947AA genotype combination was significantly associated as risk towards hypertension (OR (95% CI) = 2.40 (1.14–5.06); *p* = 0.017). Figure 2(bi,bii) compares the NLLs and HLLs population. We observed different genotype combinations of the SNPs that associated with the disease risk i.e., rs2228263CT-rs9879947GG (OR (95% CI) = 2.13 (1.09–4.16); *p* = 0.023) and protection rs2228263CC-rs9879947AG (OR (95% CI) = 0.06 (0.00–0.50); *p* = 4.78 × 10^−4^). Further, the risk-interaction models, rs2228263TT-rs9879947AA in case–control groups from HA and rs2228263CT-rs9879947GG in case–control groups from sea-level significantly correlated with high THBS1 levels (Figure 2c). The combination of risk-associated genotype rs2228263TT-rs9879947AA of THBS1 and CD47 was prevalent in HHLs group associated with high THBS1 levels when compared to its controls, NHLs (*p* = 0.002, Figure 2(ci)). For Cohort 2, the same genotype-interaction rs2228263TT-rs9879947AA depicted high risk but without any significance (Figure 2(bi)). The other significant genotype-interaction, rs2228263CT-rs9879947GG prevalent in HLLs group, was associated with high THBS1 levels compared to its controls, NLLs (*p* = 0.008, Figure 2(cii)).

### 3.6. THBS-CD Family Genes Variants Regulate Hypertension under Hypobaric Hypoxia

The THBS1 levels were comparatively greater in the HA hypertensive group as compared to the sea-level hypertensive group. Thus, to understand the genetic basis behind the increased THBS1 levels and consequently to adaptation at HA, more molecules of this family were assessed in our HA cohort.

#### 3.6.1. Variants of THBS-CD Family Genes Associate with Hypertension at HA

Genetic association study using logistic regression analysis was performed with PLINK v1.07 in the THBS-CD family genes (Table S1). At an allelic level, 122 SNPs of 34 genes were significantly associated with hypertension in Cohort 2 (*p* < 0.05 to 0.001). The detailed statistical analysis also revealed the importance of 69 SNPs towards disease protection (OR between 0.14 and 0.80 at 95% CI, Table S2). Out of these significant SNPs of THBS-CD family genes, SNP rs1438739 of *THBS4* emerged the most significant (OR (95% CI) = 0.39; *p* = 0.001). Likewise, 53 SNPs associated with the risk towards disease (OR between 1.21 and 5 at 95% CI, Table S2); here, intergenic SNP rs1006472 belonging to *CD8B2;ST6GAL2* emerged as the most significant with an OR of 1.99 (*p* = 0.002). Additionally, the other high-risk intergenic SNPs such as rs1998081 and rs2424515 of thrombomodulin (*THBD*) and *CD93* have also been found to be associated. Interestingly, there was an involvement of 3′UTR SNPs in the risk phenotype for hypertension as compared to the SNPs involved in the protection. The 3′UTR SNPs included rs7492 (OR = 4.37) and rs2749812 (OR = 3.47) of *CD93*. The other 3′UTR risk SNPs involved are rs11653281, rs11607862 and rs3733593 of *CD300C*, *CD44* and *CD38*, respectively.

#### 3.6.2. Within Gene and Gene–Gene Interactions Define Etiology of Hypertension

The 122 SNPs that emerged significantly through the genetic association study were subjected to the SNP–SNP interactions that covered both within the gene and between genes (gene–gene) interactions. It revealed 175 protective (OR 95% CI between 0.03 and 0.49) and 127 risk (OR at 95% CI between 2.12 and 47.75) associations with hypertensive (Table S3). The risk SNPs of *THBS4, CD8B2;ST6GAL2, THBS2;WDR27, CD40;CDH22* were the most prevalent in the interactions. The involvement of *CD8B2;ST6GAL2* intergenic SNPs in the risk interactions supported our single-locus genetic finding.

#### 3.6.3. Haplotypes Associate with Hypertension

The 122 SNPs provided us with the 15 significant (*p* < 0.05) haplotypes that included nine protective haplotypes (OR at 95% CI between 0.27 and 0.57) and six risk haplotypes (OR at 95% CI between 1.69 and 4.44) (Table S4). The highest odds ratio with OR (95% CI) = 4.44 was observed for the haplotype rs2424515G-rs1998081A-rs7492A-rs2749812A of *THBD;CD93* SNPs on chromosome 20; which is in accordance with our single and multiple-locus data analyses.

### 3.7. Protein–Protein Interactions Inclined toward Hypertension

**Figure 3:**
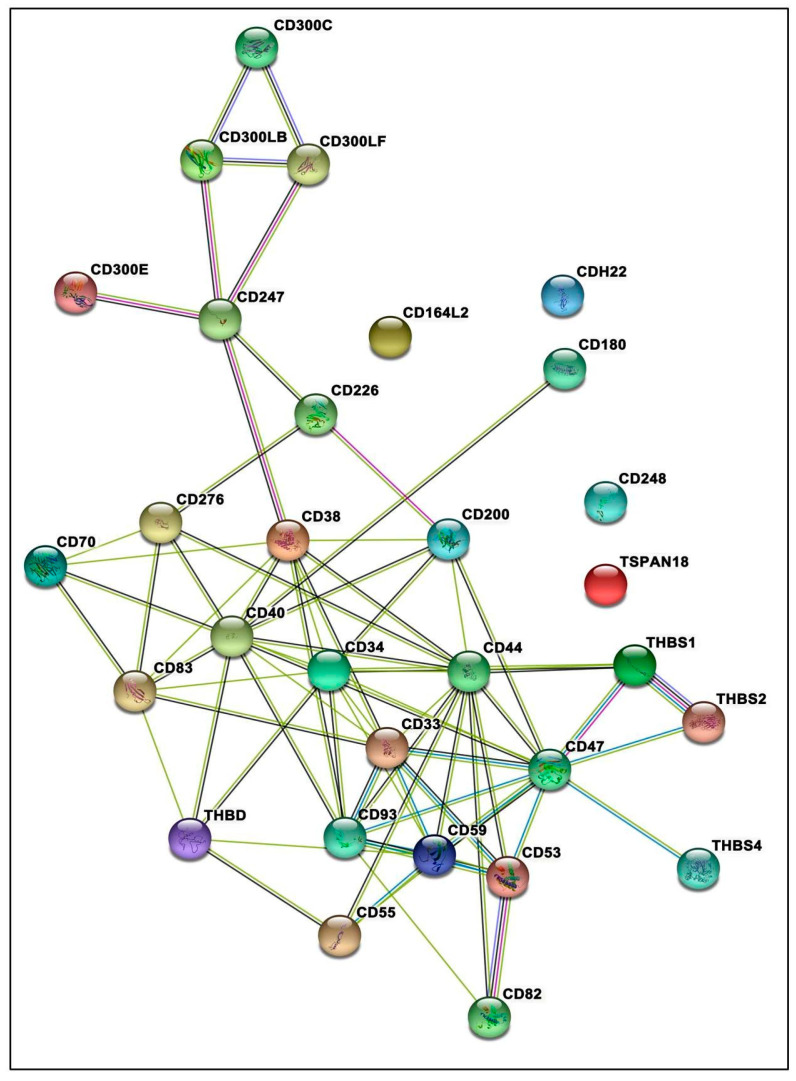
Network landscape of THBS-CD family proteins that showed significant association with hypertension. The network showed significant interactions of CD47 with THBS1, THBS2 and THBS4 and other important members of CD family having a combined confidence score > 0.89. The depicted protein–protein interactions (PPIs) support our genetic data findings by highlighting the physiological functions involved in hypertension.

STRING analysis revealed a significant network among the identified members of the THBS-CD family with a high combined confidence score (CCS; Figure 3). Among the 32 proteins, 26 proteins participated in the significant GO terms and pathways (Figure S1). Adaptive immune system (*p* = 1.71 × 10^−7^) and homeostasis (*p* < 0.05) were the two significant pathways without enrichment. Calcium ion binding was the most significantly associated molecular function (*p* = 0.006), regulation of the immune system was the most significant biological process (*p* = 4.90 × 10^−12^), and cell surface (*p* = 5.65 × 10^−11^) was the most significant cellular component involved. Based on CCS with a threshold cut-off of >0.89, we observed THBS1 and CD47 as the most important proteins showing the highest scores of 0.94 and 0.99, respectively. The CCS result implies that the affinity of CD47 towards the different isoforms of THBS is in the decreasing order of THBS1 > THBS2 > THBS4. However, the role of other CDs such as CD53 and CD59 were also found to be critical in maintaining such interactions.

## 4. Discussion

Hypertension progression involves the interactions between genetic susceptibilities and exposure to harsh environmental conditions such as the hypobaric hypoxia at HA [19,20]. The decrease in partial pressure of inspired PO_2_ and blood arterial oxygen saturation (SaO_2_) due to hypoxia adversely affect the pathways such as oxygen-sensing, renin-angiotensin–aldosterone system, and oxidative stress that are strongly implicated in various HA-related disorders [21]. However, the resident population of HA has acquired a distinct genetic setup with natural selection to adapt to the harsh climatic conditions [22]. In the process, several genetic markers associating with various pertinent pathways and diseases including hypertension have been identified [11,23,24,25]. Nonetheless, similar observations need to be ascertained for other pertinent pathways implicated in hypertension prevalent at HA. The present study conjectures the causal role of one such molecule THBS1 provoked under hypoxia and involved in the endothelial dysfunction, angiogenesis, and platelet aggregations incriminated in hypertension pathophysiology [26,27,28]. Furthermore, hypertension is associated with other risk factors such as smoking and other air pollutants that exponentially increases the risk of cardiovascular disorders [29]. Thus, the outcome of this study may be applicable to numerous pathological events where hypoxia as well as hypertension are well-established complications. The role of THBS has been primarily investigated in cancers and inflammatory disorders [30,31,32]. THBS1 was reported to contribute to the invasive behavior during glioblastoma (GBM) development through TGFβ canonical pathway [30]. Another study reported hypoxia-induced pathologic THBS1, driving TGF-β activation, and downstream non-canonical signaling via GTP-RhoA and Rho-kinase activation resulting in vasoconstriction and pulmonary hypertension [31]. A similar study demonstrated hypoxia-associated upregulation of THBS1 in the lungs due to increased HIF-2α protein expression in the pulmonary vasculature contributing to pulmonary arterial hypertension-driven vascular remodeling and vasoconstriction [32]. All of these studies established the role of hypoxia in the induction of THBS1 levels.

The present study provided relevant insights with respect to THBS1 molecule and the hypertensive patients both at hypobaric hypoxia and normoxia condition. The study Cohort 1 composed of the population of Tibeto-Burman ethnicity residing at HA are continuously exposed to the hypobaric hypoxic environment. While, the study Cohort 2 are composed of the individuals from Indo-Aryan ethnicity, who reside at sea-level. The primary observation was the significant upregulation of plasma THBS1 levels in the hypertensive groups of both cohorts. However, under the hypobaric hypoxic condition, the THBS1 levels were significantly greater in the HA hypertensive patients as compared to the sea-level hypertensive patients. The hypobaric hypoxia condition seemingly enhances THBS1 levels in the hypertensive patients residing at HA as has been depicted by our results. HA stimulates pulmonary vasoconstriction characterized by progressive changes in the pulmonary vasculature leading to the decreased blood flow and aggravation of tissue hypoxia [33]. The increased THBS1 levels may further indicate the worsening of tissue oxygen perfusion at HA because THBS1 acts as an antagonist to NO [27]. NO, an endothelium-derived relaxing factor and a known vasodilator regulates blood flow and improves oxygenation [34,35]. THBS1, in addition, contributes to pulmonary vascular remodeling through the stimulation of platelet aggregation, a state that may lead to thrombotic complications [36]. In fact, thrombosis has been identified as one of the serious cardiovascular issues at HA [37]. It, thus, signifies the importance of THBS1 at HA. Investigations in this direction are needed, though. Furthermore, THBS1 is an important endogenous inhibitor of angiogenesis by modulation of VEGF signaling that contributes to the microcirculation and capillary density, the phenomena that are again of great relevance to HA [8]. Erythropoietin, another major molecule for acclimatization during HA exposure, has effects on endothelial-derived THBS1 secretions, suggesting the potential role of THBS1 on erythroid cells [38]. Receiver operator characteristics analysis to determine the influence and association of HA on THBS1 levels further supported our observations of enhanced THBS1 levels in the hypertensive highlanders as compared to hypertensive patients from sea-level. We observed good overall discrimination on the basis of the obtained results with the significant area under curve of more than 0.6 for THBS1 levels. The clinical data in both the cohorts revealed MAP as the most significant parameter and its positive correlation with the THBS1 levels in the hypertensive groups added to its functional role. Nonetheless, the correlation between MAP and THBS1 levels in hypertensive highlanders was much greater and significant as compared to the correlation in hypertensive patients at sea-level. Next, we advanced our hypothesis to find the differential genetic variants belonging to the THBS pathway and several of its CD receptor molecules.

Our result demonstrated a differential distribution of *THBS1* rs2228263 and *CD47* rs9879947. The latter SNP was significant in Cohort 1, while the former SNP was significant in Cohort 2. Perhaps, this difference could also be attributed to the different ethnicities of the two study cohorts. Interestingly, the gene–gene interaction model highlighted the risk contribution of *THBS1* rs2228263TT/CT genotypes towards hypertension, which is also associated with the increased THBS1 levels. *THBS1* rs2228263 has been investigated in several diseases such as coronary artery disease [14], pancreatic cancer [15] and sickle cell anemia [16]. The synonymous SNP at the regulatory promoter region with a binding affinity towards proteins such as RNA Polymerase II Subunit A and chromodomain helicase DNA binding protein 1 may have a causal role. Likewise, the *CD47* rs9879947 SNP has been associated with distant metastasis in a dominant model in the colorectal cancer prognosis [17].

The enhanced THBS1 levels under hypobaric hypoxia persuaded us to further explore the contribution of the *THBS1-CD* family genes in our HA Cohort 1, and we could identify the association of 122 SNPs of 34 genes. The SNPs with the highest risk towards hypertension were intergenic SNPs rs1006472 of *CD8B2;ST6GAL2*, and rs1998081 and rs2424515 of *THBD;CD93*. We also found a significant differential distribution of the variants rs7492, rs2749812, rs11653281, rs11607862 and rs3733593 from the 3′-UTR of *CD93* that belongs to the Group XIV C-Type lectin family. This group has three other members, endosialin (*CD248*), *CLEC14A* and *THBD* that serve as the cofactors for thrombin and function as an anticoagulant [39]; however, in-depth study would ascertain their causal role in CVD pathophysiology. Furthermore, the multi-locus interactions coordinated with our single-locus genetic analysis depict the importance of *CD8B2;ST6GAL2.* While *CD8* functions in the identification of cytotoxic/suppressor T-cells that interact with MHC class I bearing targets [40]. The *ST6GAL2* encodes a sialyl transferase that is involved in several biological processes, such as cell adhesion, signal transduction, receptor activation and cancer progression [41]. We observed six haplotypes associated with hypertension. The haplotype with the highest risk alleles rs2424515G-rs1998081A-rs7492A-rs2749812A belonged to *THBD* and *CD93*. The physiological functions of genetics findings were depicted by network analysis. As expected, THBS1 and CD47 emerged as critical molecules. The results based on CCS imply that the affinity of CD47 towards the different isoforms of THBS is in the decreasing order THBS1>THBS2>THBS4, and CD47 also showed interactions with CD53 and CD59. The PPI interaction data supported our genetic data highlighting the risk role of THBD and CD93. Our findings are encouraging, albeit further validation of the variants may help identify contributing factors to the pathophysiology of hypertension and categorize potential targets for medical intervention strategies.

## 5. Conclusions

Our findings indicated the important role of THBS1 levels in patients with hypertension. There was an overall increase in the level of THBS1 in hypertension; however, the levels were significantly enhanced in the hypertensive highlanders as compared to hypertensive patients from sea-level. There was a significantly high positive correlation of MAP and THBS1 levels at HA as compared to the sea-level. The genetic analysis of *THBS1*, *CD47* and THBS-CD family members highlighted their complex role in hypertension pathophysiology based on the population ethnicities or the environment.

## Figures and Tables

**Table 1:** Demographic and clinical characteristics of the studied participants in Cohorts 1 and 2 of high-altitude and sea-level populations, respectively.

Parameters	NHLs (*n* = 183)	HHLs (*n* = 166)	*p*-Value	NLLs (*n* = 486)	HLLs (*n* = 509)	*p*-Value
**Sex**						
Male	48 (26%)	55 (33%)		363 (75%)	326 (64%)	
Female	135 (74%)	111 (67%)		123 (25%)	183 (36%)	
Clinical Characteristics						
Age, years	39.8 ± 9.0	40.9 ± 9.1	*NS*	38.0 ± 9.7	43.1 ± 7.7	<0.0001
BMI, kg/m^2^	21.5 ± 2.8	21.7 ± 2.7	*NS*	23.1 ± 2.6	24.4 ± 2.4	<0.0001
SBP, mm Hg	111.3 ± 8.33	141.6 ± 16.0	<0.0001	115.7 ± 6.0	157.1 ± 16.2	<0.0001
DBP, mm Hg	72.6 ± 7.5	88.9 ± 11.8	<0.0001	76.2 ± 5.1	96.6 ± 8.5	<0.0001
MAP, mm Hg	85.5 ± 6.9	106.5 ± 12.4	<0.0001	89.4 ± 5.1	116.8 ± 10.1	<0.0001

The data are represented as mean ± SD (standard deviation). Gender represents number of samples (percentage). Unpaired Student’s *t*-test was used to compare the given parameters controls and patients using Epi Info™ ver. 6. *p*-value ≤ 0.05 was considered statistically significant. *n*, number of samples; %, percent; *NS*, non-significant; NHLs, normotensive highlanders; HHLs, hypertensive highlanders; NLLs, normotensive lowlanders; HLLs, hypertensive lowlanders; BMI, body mass index; SBP, systolic blood pressure; DBP, diastolic blood pressure; MAP, mean arterial pressure.

**Table 2:** Genotype distribution of *THBS1* and *CD47* in hypertensive patient and control groups residing at high-altitude (HHLs and NHLs) and sea-level (HLLs and NLLs).

**High Altitude**							
**Gene (Variant Type)**	**SNP**	**Genotype/Allele**	**NHLs (*n* = 183)**	**HHLs (*n* = 166)**	**Logistic Regression Analysis**		
			***n*, (% Distribution)**		**χ^2^**	***p*-value**	**OR (95% CI)**
***THBS1***	rs2228263	TT	164 (89.6%)	153 (92.1%)	-	-	Reference
**(Synonymous)**		TC	16 (8.7%)	8 (4.8%)	2.03	0.154	0.52 (0.21–1.27)
	Co-dominant	CC	3 (1.6%)	5 (3.0%)	0.88	0.346	2.04 (0.46–9.06)
		TT + TC	180 (98.4%)	161 (97.0%)	-	-	Reference
	Recessive	CC	3 (1.6%)	5 (3.0%)	1.08	0.298	2.19 (0.49–9.67)
		T	344 (94.0%)	314 (94.6%)	-	-	Reference
	Allelic	C	22 (6.0%)	18 (5.4%)	0.04	0.834	0.93 (0.48–1.78)
***CD47***	rs9879947	GG	92 (50.2%)	80 (48.2%)	-	-	Reference
**(3′UTR)**		GA	79 (43.1%)	61 (36.7%)	0.3	0.58	0.88 (0.56–1.38)
	Co-dominant	AA	12 (6.5%)	25 (15.1%)	5.81	**0.016**	2.56(1.19–5.51)
		GG + GA	171 (93.4%)	141 (84.9%)	-	-	Reference
	Recessive	AA	12 (6.6%)	25 (15.1%)	6.96	**0.008**	2.68 (1.28–5.57)
		G	263 (71.9%)	221 (66.6%)	-	-	Reference
	Allelic	A	103 (28.1%)	111 (33.4%)	2.46	0.116	1.29 (0.93–1.79)
**Low Altitude**							
**Gene (Variant Type)**	**SNP**	**Genotype/Allele**	**NLLs (*n* = 486)**	**HLLs (*n* = 509)**	**Logistic Regression Analysis**		
			***n*, (% Distribution)**		**χ^2^**	***p*-value**	**OR (95% CI)**
***THBS1***	rs2228263	TT	412 (84.7%)	431 (84.7%)	-	-	Reference
**(Synonymous)**		TC	53 (11%)	69 (13.6%)	1.22	0.268	1.23 (0.85–1.79)
	Co-dominant	CC	21 (4.3%)	9 (1.7%)	5.06	**0.024**	0.40 (0.18–0.89)
		TT + TC	465 (95.7%)	500 (98.3%)	-	-	Reference
	Recessive	CC	21 (4.3%)	9 (1.7%)	5.38	**0.02**	0.39 (0.18–0.80)
		T	877 (90.2%)	931 (91.4%)	-	-	Reference
	Allelic	C	95 (9.8%)	87 (8.6%)	0.96	0.326	0.86 (0.63–1.16)
***CD47***	rs9879947	GG	167 (34.4%)	187 (36.8%)	-	-	Reference
**(3′UTR)**		GA	244 (50.1%)	232 (45.6%)	1.36	0.244	0.85 (0.65–1.11)
	Co-dominant	AA	75 (15.5%)	90 (17.6%)	0.12	0.729	1.06 (0.74–1.53)
		GG + GA	411 (84.5%)	419 (82.3%)	-	-	Reference
	Recessive	AA	75 (15.5%)	90 (17.7%)	0.86	0.351	1.16 (0.84–1.62)
		G	578 (59.5%)	606 (59.5%)	-	-	Reference
	Allelic	A	394 (40.5%)	412 (40.5%)	0	0.963	0.99 (0.83–1.18)

SNP, single nucleotide polymorphism; NHLs, normotensive highlanders; HHLs, hypertensive highlanders; NLLs, normotensive lowlanders; HLLs, hypertensive lowlanders; THBS1, Thrombospondin-1; OR (95% CI), odds ratio at 95% confidence interval; *n*, number of samples; %, percent.

## Data Availability

The data presented in this study are available with the manuscript and in the Supplementary Materials Tables S1 and S2.

## Supplementary Materials

The following are available online at https://www.mdpi.com/article/10.3390/life11090893/s1, Table S1: SNPs of THBS-CD family genes selected for genotyping, Table S2: The significant SNPs of THBS-CD family genes genotyped in hypertensive and normotensive highlanders, Table S3: Significant SNP-SNP interactions of THBS-CD family genes identified between the controls and patients of Cohort 2, Table S4: Significant haplotypes of THBS-CD family genes identified between the controls Figure S1: Significant reactome pathways, molecular functions, cellular components, gene mapping, biological processes and string interactions as identified through STRING analysis.
