# Supplementary material for: Hypertensive Patients Exhibit Enhanced Thrombospondin-1 Levels at High-Altitude
Source: Life (Basel). 2021 Aug 29;11(9):893. doi: 10.3390/life11090893 (PMC8469964; doi:10.3390/life11090893)
Supplement: Supplementary file 1 [file life-11-00893-s001.zip › life-1356239-supplementary.pdf]

life

Supplementary material of Hypertensive Patients Exhibit Enhanced Thrombospondin-1 Levels at High-Altitude

Table S1. SNPs of THBS-CD family genes selected for genotyping.

CHR	Location	SNP	Consequence	*Gene ID*	Gene Description
5	79288995	rs1438739	intronic	*THBS4*	Thrombospondin-4
2	1.07E+08	rs1006472	intergenic	*CD8B2;ST6GAL2*	T-Cell Surface Glycoprotein CD8 Beta-2 Chain ; Beta-galactoside alpha-2,6-sialyltransferase 2
2	1.07E+08	rs898456	intergenic	*CD8B2;ST6GAL2*	T-Cell Surface Glycoprotein CD8 Beta-2 Chain ; Beta-galactoside alpha-2,6-sialyltransferase 2
2	1.07E+08	rs2377685	intergenic	*CD8B2;ST6GAL2*	T-Cell Surface Glycoprotein CD8 Beta-2 Chain ; Beta-galactoside alpha-2,6-sialyltransferase 2
2	1.07E+08	rs5833209	intergenic	*CD8B2;ST6GAL2*	T-Cell Surface Glycoprotein CD8 Beta-2 Chain ; Beta-galactoside alpha-2,6-sialyltransferase 2
20	44771565	rs11696696	intergenic	*CD40;CDH22*	Tumor necrosis factor receptor superfamily member 5; Cadherin-22
20	44769666	rs6074037	intergenic	*CD40;CDH22*	Tumor necrosis factor receptor superfamily member 5; Cadherin-22
11	35213126	rs7934770	intronic	*CD44*	CD44 antigen
3	1.12E+08	rs2633610	intergenic	*CD200;BTLA*	OX-2 membrane glycoprotein; B- and T-lymphocyte attenuator
4	15874104	rs2531166	intergenic	*CD38;FGFBP1*	ADP-ribosyl cyclase/cyclic ADP-ribose hydrolase 1; Fibroblast growth factor-binding protein 1
20	44796128	rs2425759	intergenic	*CD40;CDH22*	Tumor necrosis factor receptor superfamily member 5; Cadherin-22
2	1.07E+08	rs56018727	intergenic	*CD8B2;ST6GAL2*	T-Cell Surface Glycoprotein CD8 Beta-2 Chain ; Beta-galactoside alpha-2,6-sialyltransferase 2
1	2.08E+08	rs2184820	intergenic	*CD34;PLXNA2*	Hematopoietic progenitor cell antigen CD34; Plexin-A2
20	44800445	rs2425764	intergenic	*CD40;CDH22*	Tumor necrosis factor receptor superfamily member 5; Cadherin-22
5	79292899	rs7707036	intronic	*THBS4*	Thrombospondin-4
17	72536901	rs4788845	downstream	*CD300C*	CMRF35-like molecule 6
2	1.07E+08	rs74879177	intergenic	*CD8B2;ST6GAL2*	T-Cell Surface Glycoprotein CD8 Beta-2 Chain ; Beta-galactoside alpha-2,6-sialyltransferase 2
3	1.12E+08	rs2633611	intergenic	*CD200;BTLA*	OX-2 membrane glycoprotein; B- and T-lymphocyte attenuator
11	35210798	rs996076	intronic	*CD44*	CD44 antigen
11	44654168	rs7113690	intergenic	*CD82;TSPAN18*	CD82 antigen; Tetraspanin-18
2	1.07E+08	rs6721068	intergenic	*CD8B2;ST6GAL2*	T-Cell Surface Glycoprotein CD8 Beta-2 Chain ; Beta-galactoside alpha-2,6-sialyltransferase 2
17	72649890	rs35729709	intergenic	*CD300E;RAB37*	CMRF35-like molecule 2; Ras-related protein Rab-37
1	2.08E+08	rs12095775	intergenic	*CD34;PLXNA2*	Hematopoietic progenitor cell antigen CD34; Plexin-A2
17	72532409	rs4789074	intergenic	*CD300LB;CD300C*	CMRF35-like molecule 7; CMRF35-like molecule 6
2	1.07E+08	rs2241992	intergenic	*CD8B2;ST6GAL2*	T-Cell Surface Glycoprotein CD8 Beta-2 Chain ; Beta-galactoside alpha-2,6-sialyltransferase 2
2	1.07E+08	rs1448118	intergenic	*CD8B2;ST6GAL2*	T-Cell Surface Glycoprotein CD8 Beta-2 Chain ; Beta-galactoside alpha-2,6-sialyltransferase 2

CHR	Location	SNP	Consequence	*Gene ID*	Gene Description
5	66752829	rs1274868	intergenic	*CD180;LINC02242*	CD180 antigen; Long Intergenic Non-Protein Coding RNA 2242
11	44654385	rs7113954	intergenic	*CD82;TSPAN18*	CD82 antigen; Tetraspanin-18
2	1.07E+08	rs4381779	intergenic	*CD8B2;ST6GAL2*	T-Cell Surface Glycoprotein CD8 Beta-2 Chain ; Beta-galactoside alpha-2,6-sialyltransferase 2
1	2.08E+08	rs6666666	intergenic	*CD34;PLXNA2*	Hematopoietic progenitor cell antigen CD34; Plexin-A2
3	1.12E+08	rs2633568	intergenic	*CD200;BTLA*	OX-2 membrane glycoprotein; B- and T-lymphocyte attenuator
20	23060257	rs7492	UTR3	*CD93*	Complement component C1q receptor
3	1.12E+08	rs721363	intergenic	*CD200;BTLA*	OX-2 membrane glycoprotein; B- and T-lymphocyte attenuator
6	14187776	rs9370732	intergenic	*CD83;LINC01108*	CD83 antigen; Long Intergenic Non-Protein Coding RNA 1108
1	1.68E+08	rs199582811	intergenic	*CD247;CREG1*	T-cell surface glycoprotein CD3 zeta chain; Protein CREG1
11	44649802	rs11820438	intergenic	*CD82;TSPAN18*	CD82 antigen; Tetraspanin-18
6	1.7E+08	rs6605523	intergenic	*THBS2;WDR27*	Thrombospondin-2; WD repeat-containing protein 27
1	2.08E+08	rs3754171	intergenic	*CD34;PLXNA2*	Hematopoietic progenitor cell antigen CD34; Plexin-A2
1	2.08E+08	rs3754172	intergenic	*CD34;PLXNA2*	Hematopoietic progenitor cell antigen CD34; Plexin-A2
1	2.08E+08	rs2267894	intergenic	*CD34;PLXNA2*	Hematopoietic progenitor cell antigen CD34; Plexin-A2
2	1.07E+08	rs1006413	intergenic	*CD8B2;ST6GAL2*	T-Cell Surface Glycoprotein CD8 Beta-2 Chain ; Beta-galactoside alpha-2,6-sialyltransferase 2
11	66093612	rs1892938	intergenic	*CD248;RIN1*	Endosialin; Ras and Rab interactor 1
2	1.07E+08	rs1551718	intergenic	*CD8B2;ST6GAL2*	T-Cell Surface Glycoprotein CD8 Beta-2 Chain ; Beta-galactoside alpha-2,6-sialyltransferase 2
1	1.11E+08	rs7521468	intergenic	*CD53;LRIF1*	Leukocyte surface antigen CD53; Ligand-dependent nuclear receptor-interacting factor 1
2	1.07E+08	rs12473520	intergenic	*CD8B2;ST6GAL2*	T-Cell Surface Glycoprotein CD8 Beta-2 Chain ; Beta-galactoside alpha-2,6-sialyltransferase 2
1	1.11E+08	rs1936942	intergenic	*CD53;LRIF1*	Leukocyte surface antigen CD53; Ligand-dependent nuclear receptor-interacting factor 1
17	72533076	rs11652446	intergenic	*CD300LB;CD300C*	CMRF35-like molecule 7; CMRF35-like molecule 6
17	72523224	rs10512596	intronic	*CD300LB*	CMRF35-like molecule 7
4	15909690	rs13103381	intergenic	*CD38;FGFBP1*	ADP-ribosyl cyclase/cyclic ADP-ribose hydrolase 1; Fibroblast growth factor-binding protein 1
4	15910385	rs2532070	intergenic	*CD38;FGFBP1*	ADP-ribosyl cyclase/cyclic ADP-ribose hydrolase 1; Fibroblast growth factor-binding protein 1
15	73987730	rs80307614	intronic	*CD276*	CD276 antigen
3	1.12E+08	rs2705545	intergenic	*CD200;BTLA*	OX-2 membrane glycoprotein; B- and T-lymphocyte attenuator
2	1.07E+08	rs4487086	intergenic	*CD8B2;ST6GAL2*	T-Cell Surface Glycoprotein CD8 Beta-2 Chain ; Beta-galactoside alpha-2,6-sialyltransferase 2
20	23057482	rs1998081	intergenic	*THBD;CD93*	Thrombomodulin; Complement component C1q receptor
20	23048733	rs2424515	intergenic	*THBD;CD93*	Thrombomodulin; Complement component C1q receptor
11	33752403	rs1738548	intronic	*CD59*	CD59 glycoprotein

CHR	Location	SNP	Consequence	*Gene ID*	Gene Description
1	1.11E+08	rs1282023	intergenic	*CD53;LRIF1*	Leukocyte surface antigen CD53; Ligand-dependent nuclear receptor-interacting factor 1
1	1.11E+08	rs12077952	intergenic	*CD53;LRIF1*	Leukocyte surface antigen CD53; Ligand-dependent nuclear receptor-interacting factor 1
2	1.07E+08	rs4676262	intergenic	*CD8B2;ST6GAL2*	T-Cell Surface Glycoprotein CD8 Beta-2 Chain ; Beta-galactoside alpha-2,6-sialyltransferase 2
1	1.11E+08	rs11102175	intronic	*CD53*	Leukocyte surface antigen CD53
1	2.08E+08	rs76643201	intergenic	*CD34;PLXNA2*	Hematopoietic progenitor cell antigen CD34; Plexin-A2
20	23062927	rs2749812	UTR3	*CD93*	Complement component C1q receptor
3	1.12E+08	rs11708711	intergenic	*CD200;BTLA*	OX-2 membrane glycoprotein; B- and T-lymphocyte attenuator
11	44639551	rs9971373	intronic	*CD82*	CD82 antigen
2	1.07E+08	rs12712257	intergenic	*CD8B2;ST6GAL2*	T-Cell Surface Glycoprotein CD8 Beta-2 Chain ; Beta-galactoside alpha-2,6-sialyltransferase 2
2	1.07E+08	rs7589737	intergenic	*CD8B2;ST6GAL2*	T-Cell Surface Glycoprotein CD8 Beta-2 Chain ; Beta-galactoside alpha-2,6-sialyltransferase 2
1	27707859	rs17162558	intronic	*CD164L2*	CD164 molecule like 2
17	72691263	rs34303409	exonic	*CD300LF*	CMRF35-like molecule 1
2	1.07E+08	rs10514794	intergenic	*CD8B2;ST6GAL2*	T-Cell Surface Glycoprotein CD8 Beta-2 Chain ; Beta-galactoside alpha-2,6-sialyltransferase 2
12	6560573	rs1059501	ncRNA_exonic	*CD27-AS1*	CD27 Antisense RNA 1
3	1.12E+08	rs721361	intergenic	*CD200;BTLA*	OX-2 membrane glycoprotein; B- and T-lymphocyte attenuator
1	27705565	rs2474287	downstream	*CD164L2*	CD164 molecule like 2
17	72537245	rs11653281	UTR3	*CD300C*	CMRF35-like molecule 6
3	1.12E+08	rs13097590	intergenic	*CD200;BTLA*	OX-2 membrane glycoprotein; B- and T-lymphocyte attenuator
11	35238459	rs1547059	intronic	*CD44*	CD44 antigen
11	35223945	rs713330	intronic	*CD44*	CD44 antigen
11	35217745	rs10836340	intronic	*CD44*	CD44 antigen
11	35226155	rs9666607	exonic	*CD44*	CD44 antigen
20	44757213	rs3765457	intronic	*CD40*	Tumor necrosis factor receptor superfamily member 5
5	79295243	rs12054697	intronic	*THBS4*	Thrombospondin-4
1	1.11E+08	rs3790722	intronic	*CD53*	Leukocyte surface antigen CD53
1	1.67E+08	rs1003815	intergenic	*CD247;CREG1*	T-cell surface glycoprotein CD3 zeta chain; Protein CREG1
19	51752217	rs12971800	intergenic	*CD33;SIGLECL1*	Myeloid cell surface antigen CD33; SIGLEC family-like protein 1
18	67568836	rs1788110	intronic	*CD226*	CD226 antigen
19	51727962	rs3865444	upstream	*CD33*	Myeloid cell surface antigen CD33
11	44590729	rs10769059	intronic	*CD82*	CD82 antigen

CHR	Location	SNP	Consequence	Gene ID	Gene Description
11	44652154	rs10769062	intergenic	*CD82;TSPAN18*	CD82 antigen; Tetraspanin-18
5	66694360	rs1812472	intergenic	*CD180;LINC02242*	CD180 antigen; Long Intergenic Non-Protein Coding RNA 2242
5	66714141	rs186460	intergenic	*CD180;LINC02242*	CD180 antigen; Long Intergenic Non-Protein Coding RNA 2242
5	66705097	rs317976	intergenic	*CD180;LINC02242*	CD180 antigen; Long Intergenic Non-Protein Coding RNA 2242
11	44615815	rs12576112	intronic	*CD82*	CD82 antigen
11	44596817	rs17541041	intronic	*CD82*	CD82 antigen
4	15784598	rs3756243	intronic	*CD38*	ADP-ribosyl cyclase/cyclic ADP-ribose hydrolase 1
1	2.08E+08	rs2490255	intergenic	*CD55;CR2*	Complement decay-accelerating factor; Complement receptor type 2
19	6609480	rs73922419	intergenic	*CD70;TNFSF14*	CD70 antigen; Tumor necrosis factor ligand superfamily member 14
11	35210866	rs996075	intronic	*CD44*	CD44 antigen
4	15906804	rs12513168	intergenic	*CD38;FGFBP1*	ADP-ribosyl cyclase/cyclic ADP-ribose hydrolase 1; Fibroblast growth factor-binding protein 1
4	15907133	rs11944611	intergenic	*CD38;FGFBP1*	ADP-ribosyl cyclase/cyclic ADP-ribose hydrolase 1; Fibroblast growth factor-binding protein 1
18	67565898	rs1788112	intronic	*CD226*	CD226 antigen
2	1.07E+08	rs7599975	intergenic	*CD8B2;ST6GAL2*	T-Cell Surface Glycoprotein CD8 Beta-2 Chain ; Beta-galactoside alpha-2,6-sialyltransferase 2
4	15818391	rs3796868	intronic	*CD38*	ADP-ribosyl cyclase/cyclic ADP-ribose hydrolase 1
2	1.07E+08	rs17220280	intergenic	*CD8B2;ST6GAL2*	T-Cell Surface Glycoprotein CD8 Beta-2 Chain ; Beta-galactoside alpha-2,6-sialyltransferase 2
11	44691479	rs7935663	intergenic	*CD82;TSPAN18*	CD82 antigen; Tetraspanin-18
11	44657500	rs12420889	intergenic	*CD82;TSPAN18*	CD82 antigen; Tetraspanin-18
20	44784622	rs6032681	intergenic	*CD40;CDH22*	Tumor necrosis factor receptor superfamily member 5; Cadherin-22
11	44649231	rs4755854	intergenic	*CD82;TSPAN18*	CD82 antigen; Tetraspanin-18
12	6557674	rs2253610	ncRNA_intronic	*CD27-AS1*	CD27 Antisense RNA 1
11	44645525	rs11038084	intergenic	*CD82;TSPAN18*	CD82 antigen; Tetraspanin-18
3	1.12E+08	rs13090873	intergenic	*CD200;BTLA*	OX-2 membrane glycoprotein; B- and T-lymphocyte attenuator
11	35244574	rs11607862	UTR3	*CD44*	CD44 antigen
20	44755889	rs11569333	intronic	*CD40*	Tumor necrosis factor receptor superfamily member 5
11	35242660	rs2065004	intronic	*CD44*	CD44 antigen
2	1.07E+08	rs6543438	intergenic	*CD8B2;ST6GAL2*	T-Cell Surface Glycoprotein CD8 Beta-2 Chain ; Beta-galactoside alpha-2,6-sialyltransferase 2
4	15854725	rs3733593	UTR3	*CD38*	ADP-ribosyl cyclase/cyclic ADP-ribose hydrolase 1
4	15856799	rs13136270	intergenic	*CD38;FGFBP1*	ADP-ribosyl cyclase/cyclic ADP-ribose hydrolase 1; Fibroblast growth factor-binding protein 1
11	44596428	rs117170259	intronic	*CD82*	CD82 antigen

CHR	Location	SNP	Consequence	*Gene ID*	Gene Description
20	44763201	rs62215622	intergenic	*CD40;CDH22*	Tumor necrosis factor receptor superfamily member 5; Cadherin-22
6	1.7E+08	rs1028296	intergenic	*THBS2;WDR27*	Thrombospondin-2; WD repeat-containing protein 27
11	33753021	rs2231454	intronic	*CD59*	CD59 glycoprotein
19	6654011	rs13343619	intergenic	*CD70;TNFSF14*	CD70 antigen; Tumor necrosis factor ligand superfamily member 14
3	1.12E+08	rs13091125	intergenic	*CD200;BTLA*	OX-2 membrane glycoprotein; B- and T-lymphocyte attenuator
11	44688577	rs10838327	intergenic	*CD82;TSPAN18*	CD82 antigen; Tetraspanin-18

The selected SNPs are from the optimized tag SNP content with 1000 Genomes Project pilot data. SNP; Single nucleotide polymorphism; CHR, chromosome.

Table S2. The significant SNPs of THBS-CD family genes genotyped in hypertensive and normotensive highlanders.

CHR	Location	SNP	OR	*P* value	Consequence	Gene information
5	79288995	rs1438739	0.3944	0.001044	intronic	*THBS4*
2	1.07E+08	rs1006472	1.994	0.002112	intergenic	*CD8B2;ST6GAL2*
2	1.07E+08	rs898456	1.994	0.002112	intergenic	*CD8B2;ST6GAL2*
2	1.07E+08	rs2377685	1.994	0.002112	intergenic	*CD8B2;ST6GAL2*
2	1.07E+08	rs5833209	1.994	0.002112	intergenic	*CD8B2;ST6GAL2*
20	44771565	rs11696696	0.503	0.00228	intergenic	*CD40;CDH22*
20	44769666	rs6074037	0.556	0.003193	intergenic	*CD40;CDH22*
11	35213126	rs7934770	0.5908	0.003592	intronic	*CD44*
3	1.12E+08	rs2633610	0.6923	0.003829	intergenic	*CD200;BTLA*
4	15874104	rs2531166	0.6342	0.0052	intergenic	*CD38;FGFBP1*
20	44796128	rs2425759	0.3444	0.005945	intergenic	*CD40;CDH22*
2	1.07E+08	rs56018727	0.3362	0.006136	intergenic	*CD8B2;ST6GAL2*
1	2.08E+08	rs2184820	0.5199	0.006937	intergenic	*CD34;PLXNA2*
20	44800445	rs2425764	0.3968	0.007091	intergenic	*CD40;CDH22*
5	79292899	rs7707036	0.54	0.007375	intronic	*THBS4*
17	72536901	rs4788845	2.009	0.00819	downstream	*CD300C*
2	1.07E+08	rs74879177	0.236	0.008406	intergenic	*CD8B2;ST6GAL2*
3	1.12E+08	rs2633611	0.7088	0.009061	intergenic	*CD200;BTLA*
11	35210798	rs996076	0.6663	0.009102	intronic	*CD44*
11	44654168	rs7113690	0.5571	0.009289	intergenic	*CD82;TSPAN18*
2	1.07E+08	rs6721068	0.5386	0.0101	intergenic	*CD8B2;ST6GAL2*
17	72649890	rs35729709	0.6708	0.01067	intergenic	*CD300E;RAB37*
1	2.08E+08	rs12095775	0.4381	0.01134	intergenic	*CD34;PLXNA2*
17	72532409	rs4789074	2.281	0.01135	intergenic	*CD300LB;CD300C*
2	1.07E+08	rs2241992	0.5405	0.01159	intergenic	*CD8B2;ST6GAL2*
2	1.07E+08	rs1448118	1.814	0.01231	intergenic	*CD8B2;ST6GAL2*
5	66752829	rs1274868	0.1451	0.01238	intergenic	*CD180;LINC02242*
11	44654385	rs7113954	0.5636	0.01273	intergenic	*CD82;TSPAN18*
2	1.07E+08	rs4381779	0.4181	0.01286	intergenic	*CD8B2;ST6GAL2*

CHR	Location	SNP	OR	*P* value	Consequence	Gene information
1	2.08E+08	rs6666666	0.4456	0.01326	intergenic	*CD34;PLXNA2*
3	1.12E+08	rs2633568	0.7703	0.01384	intergenic	*CD200;BTLA*
20	23060257	rs7492	4.37	0.01391	UTR3	*CD93*
3	1.12E+08	rs721363	0.727	0.01435	intergenic	*CD200;BTLA*
6	14187776	rs9370732	3.131	0.01524	intergenic	*CD83;LINC01108*
1	1.68E+08	rs199582811	0.6481	0.01573	intergenic	*CD247;CREG1*
11	44649802	rs11820438	1.645	0.01582	intergenic	*CD82;TSPAN18*
6	1.7E+08	rs6605523	2.183	0.01599	intergenic	*THBS2;WDR27*
1	2.08E+08	rs3754171	0.4786	0.01647	intergenic	*CD34;PLXNA2*
1	2.08E+08	rs3754172	0.4786	0.01647	intergenic	*CD34;PLXNA2*
1	2.08E+08	rs2267894	0.4786	0.01647	intergenic	*CD34;PLXNA2*
2	1.07E+08	rs1006413	1.76	0.01713	intergenic	*CD8B2;ST6GAL2*
11	66093612	rs1892938	0.616	0.01771	intergenic	*CD248;RIN1*
2	1.07E+08	rs1551718	1.654	0.01777	intergenic	*CD8B2;ST6GAL2*
1	1.11E+08	rs7521468	1.352	0.01822	intergenic	*CD53;LRIF1*
2	1.07E+08	rs12473520	1.767	0.01869	intergenic	*CD8B2;ST6GAL2*
1	1.11E+08	rs1936942	1.256	0.01905	intergenic	*CD53;LRIF1*
17	72533076	rs11652446	2.058	0.01908	intergenic	*CD300LB;CD300C*
17	72523224	rs10512596	2.058	0.01908	intronic	*CD300LB*
4	15909690	rs13103381	1.476	0.02006	intergenic	*CD38;FGFBP1*
4	15910385	rs2532070	1.476	0.02006	intergenic	*CD38;FGFBP1*
15	73987730	rs80307614	2.079	0.02022	intronic	*CD276*
3	1.12E+08	rs2705545	0.7871	0.02091	intergenic	*CD200;BTLA*
2	1.07E+08	rs4487086	0.5877	0.02121	intergenic	*CD8B2;ST6GAL2*
20	23057482	rs1998081	5	0.02179	intergenic	*THBD;CD93*
20	23048733	rs2424515	5	0.02179	intergenic	*THBD;CD93*
11	33752403	rs1738548	1.526	0.0221	intronic	*CD59*
1	1.11E+08	rs1282023	1.219	0.02227	intergenic	*CD53;LRIF1*
1	1.11E+08	rs12077952	1.219	0.02227	intergenic	*CD53;LRIF1*
2	1.07E+08	rs4676262	1.745	0.02309	intergenic	*CD8B2;ST6GAL2*
1	1.11E+08	rs11102175	0.6748	0.02405	intronic	*CD53*
1	2.08E+08	rs76643201	0.4643	0.02458	intergenic	*CD34;PLXNA2*
20	23062927	rs2749812	3.47	0.02523	UTR3	*CD93*
3	1.12E+08	rs11708711	0.6937	0.02598	intergenic	*CD200;BTLA*
11	44639551	rs9971373	3.722	0.02768	intronic	*CD82*
2	1.07E+08	rs12712257	0.6327	0.02773	intergenic	*CD8B2;ST6GAL2*
2	1.07E+08	rs7589737	1.615	0.02874	intergenic	*CD8B2;ST6GAL2*
1	27707859	rs17162558	2.159	0.03042	intronic	*CD164L2*
17	72691263	rs34303409	0.3857	0.03055	exonic	*CD300LF*
2	1.07E+08	rs10514794	0.6092	0.03099	intergenic	*CD8B2;ST6GAL2*

CHR	Location	SNP	OR	*P* value	Consequence	Gene information
12	6560573	rs1059501	0.6385	0.03108	ncRNA_exonic	*CD27-AS1*
3	1.12E+08	rs721361	0.8095	0.03151	intergenic	*CD200;BTLA*
1	27705565	rs2474287	1.798	0.03182	downstream	*CD164L2*
17	72537245	rs11653281	1.879	0.03262	UTR3	*CD300C*
3	1.12E+08	rs13097590	0.7493	0.03284	intergenic	*CD200;BTLA*
11	35238459	rs1547059	0.3665	0.03288	intronic	*CD44*
11	35223945	rs713330	0.68	0.03315	intronic	*CD44*
11	35217745	rs10836340	0.68	0.03315	intronic	*CD44*
11	35226155	rs9666607	0.68	0.03315	exonic	*CD44*
20	44757213	rs3765457	0.1937	0.03329	intronic	*CD40*
5	79295243	rs12054697	0.7057	0.03411	intronic	*THBS4*
1	1.11E+08	rs3790722	0.7031	0.03495	intronic	*CD53*
1	1.67E+08	rs1003815	0.4156	0.035	intergenic	*CD247;CREG1*
19	51752217	rs12971800	1.545	0.03512	intergenic	*CD33;SIGLECL1*
18	67568836	rs1788110	0.5971	0.03535	intronic	*CD226*
19	51727962	rs3865444	1.536	0.03536	upstream	*CD33*
11	44590729	rs10769059	1.771	0.03561	intronic	*CD82*
11	44652154	rs10769062	1.508	0.03615	intergenic	*CD82;TSPAN18*
5	66694360	rs1812472	0.3438	0.03639	intergenic	*CD180;LINC02242*
5	66714141	rs186460	0.3438	0.03639	intergenic	*CD180;LINC02242*
5	66705097	rs317976	0.3438	0.03639	intergenic	*CD180;LINC02242*
11	44615815	rs12576112	3.87	0.03717	intronic	*CD82*
11	44596817	rs17541041	3.87	0.03717	intronic	*CD82*
4	15784598	rs3756243	0.5655	0.03761	intronic	*CD38*
1	2.08E+08	rs2490255	1.513	0.03769	intergenic	*CD55;CR2*
19	6609480	rs73922419	2.87	0.03824	intergenic	*CD70;TNFSF14*
11	35210866	rs996075	0.7838	0.03923	intronic	*CD44*
4	15906804	rs12513168	1.332	0.03931	intergenic	*CD38;FGFBP1*
4	15907133	rs11944611	1.332	0.03931	intergenic	*CD38;FGFBP1*
18	67565898	rs1788112	0.4741	0.03931	intronic	*CD226*
2	1.07E+08	rs7599975	0.6545	0.03963	intergenic	*CD8B2;ST6GAL2*
4	15818391	rs3796868	1.879	0.04017	intronic	*CD38*
2	1.07E+08	rs17220280	0.42	0.04024	intergenic	*CD8B2;ST6GAL2*
11	44691479	rs7935663	0.5455	0.04054	intergenic	*CD82;TSPAN18*
11	44657500	rs12420889	0.625	0.04084	intergenic	*CD82;TSPAN18*
20	44784622	rs6032681	0.6839	0.04103	intergenic	*CD40;CDH22*
11	44649231	rs4755854	1.604	0.04134	intergenic	*CD82;TSPAN18*
12	6557674	rs2253610	1.656	0.04199	ncRNA_intronic	*CD27-AS1*
11	44645525	rs11038084	2.421	0.04211	intergenic	*CD82;TSPAN18*
3	1.12E+08	rs13090873	0.7493	0.04286	intergenic	*CD200;BTLA*

CHR	Location	SNP	OR	*P* value	Consequence	Gene information
11	35244574	rs11607862	1.656	0.04301	UTR3	*CD44*
20	44755889	rs11569333	0.2092	0.04367	intronic	*CD40*
11	35242660	rs2065004	0.4063	0.04371	intronic	*CD44*
2	1.07E+08	rs6543438	4.459	0.04441	intergenic	*CD8B2;ST6GAL2*
4	15854725	rs3733593	1.62	0.04471	UTR3	*CD38*
4	15856799	rs13136270	1.62	0.04471	intergenic	*CD38;FGFBP1*
11	44596428	rs117170259	3.29	0.04581	intronic	*CD82*
20	44763201	rs62215622	0.5234	0.04615	intergenic	*CD40;CDH22*
6	1.7E+08	rs1028296	2.782	0.04678	intergenic	*THBS2;WDR27*
11	33753021	rs2231454	0.3877	0.0472	intronic	*CD59*
19	6654011	rs13343619	0.4421	0.04789	intergenic	*CD70;TNFSF14*
3	1.12E+08	rs13091125	0.7289	0.04812	intergenic	*CD200;BTLA*
11	44688577	rs10838327	0.5619	0.04849	intergenic	*CD82;TSPAN18*

The SNPs were genotyped using Fluidigm 48.48 SNPtype assay in 69 normotensive highlanders (NHLs) and 65 hypertensive highlanders (HHLs). *PLINK* v1.07 *tool was used to perform* logistic regression analysis under a log-additive model. Significance of the SNPs was maintained at $P < 0.05$. SNP; Single nucleotide polymorphism; CHR, chromosome; OR, Odds ratio at 95% confidence interval.

Table S3. Significant SNP-SNP interactions of THBS-CD family genes identified between the controls and patients of Cohort 2.

CHR1	rsID	Gene ID	CHR2	rsID	Gene ID	OR	P
1	rs2474287	*CD164L2*	4	rs2531166	*CD38;FGFBP1*	3.287	0.02815
			4	rs12513168	*CD38;FGFBP1*	0.3345	0.01518
			4	rs11944611	*CD38;FGFBP1*	0.3345	0.01518
			4	rs13103381	*CD38;FGFBP1*	0.4081	0.04971
			4	rs2532070	*CD38;FGFBP1*	0.4081	0.04971
			11	rs1738548	*CD59*	3.833	0.0401
			12	rs1059501	*CD27-AS1*	3.679	0.01495
			18	rs1788112	*CD226*	0.2408	0.04339
1	rs3790722	*CD53*	19	rs3865444	*CD33*	0.3692	0.01944
1	rs11102175	*CD53*	11	rs7113690	*CD82;TSPAN18*	0.3099	0.03082
			11	rs7113954	*CD82;TSPAN18*	0.313	0.0322
			19	rs3865444	*CD33*	0.3886	0.02588
			19	rs12971800	*CD33;SIGLECL1*	4.664	0.03756
1	rs199582811	*CD247;CREG1*	2	rs12712257	*CD8B2;ST6GAL2*	0.3481	0.03804
			2	rs1006472	*CD8B2;ST6GAL2*	3.883	0.0143
			2	rs7599975	*CD8B2;ST6GAL2*	0.2859	0.01959
			2	rs898456	*CD8B2;ST6GAL2*	3.736	0.01682
			2	rs1551718	*CD8B2;ST6GAL2*	2.977	0.03448
			2	rs6721068	*CD8B2;ST6GAL2*	0.3398	0.04706
			2	rs2377685	*CD8B2;ST6GAL2*	3.736	0.01682
			2	rs5833209	*CD8B2;ST6GAL2*	3.736	0.01682

CHR1	rsID	Gene ID	CHR2	rsID	Gene ID	OR	P
			2	rs2241992	*CD8B2;ST6GAL2*	0.284	0.02987
			11	rs12420889	*CD82;TSPAN18*	0.2068	0.01445
1	rs2490255	*CD55;CR2*	1	rs12095775	*CD34;PLXNA2*	3.447	0.04302
			1	rs6666666	*CD34;PLXNA2*	3.448	0.04299
			3	rs11708711	*CD200;BTLA*	0.4427	0.03064
			11	rs11820438	*CD82;TSPAN18*	0.4042	0.0227
1	rs2267894	*CD34;PLXNA2*	11	rs11820438	*CD82;TSPAN18*	3.963	0.03231
			11	rs7113690	*CD82;TSPAN18*	0.1667	0.01978
			11	rs7113954	*CD82;TSPAN18*	0.1618	0.01801
			11	rs12420889	*CD82;TSPAN18*	0.2033	0.0438
1	rs3754172	*CD34;PLXNA2*	11	rs11820438	*CD82;TSPAN18*	3.963	0.03231
			11	rs7113690	*CD82;TSPAN18*	0.1667	0.01978
			11	rs7113954	*CD82;TSPAN18*	0.1618	0.01801
			11	rs12420889	*CD82;TSPAN18*	0.2033	0.0438
1	rs3754171	*CD34;PLXNA2*	11	rs11820438	*CD82;TSPAN18*	3.963	0.03231
			11	rs7113690	*CD82;TSPAN18*	0.1667	0.01978
			11	rs7113954	*CD82;TSPAN18*	0.1618	0.01801
			11	rs12420889	*CD82;TSPAN18*	0.2033	0.0438
1	rs12095775	*CD34;PLXNA2*	11	rs7113690	*CD82;TSPAN18*	0.186	0.02814
			11	rs7113954	*CD82;TSPAN18*	0.1808	0.0258
1	rs6666666	*CD34;PLXNA2*	11	rs11820438	*CD82;TSPAN18*	3.649	0.04904
			11	rs7113690	*CD82;TSPAN18*	0.1811	0.02588
			11	rs7113954	*CD82;TSPAN18*	0.1759	0.02366
1	rs2184820	*CD34;PLXNA2*	11	rs7113690	*CD82;TSPAN18*	0.2069	0.03519
			11	rs7113954	*CD82;TSPAN18*	0.1769	0.02415
			17	rs11653281	*CD300C*	3.467	0.04198
			20	rs2425759	*CD40;CDH22*	5.742	0.03347
2	rs7589737	*CD8B2;ST6GAL2*	3	rs721361	*CD200;BTLA*	0.2591	0.03185
			3	rs721363	*CD200;BTLA*	0.2471	0.02774
			11	rs996076	*CD44*	0.2714	0.04675
			11	rs9971373	*CD82*	0.09355	0.03374
			15	rs80307614	*CD276*	0.09404	0.02054
2	rs12473520	*CD8B2;ST6GAL2*	2	rs1006472	*CD8B2;ST6GAL2*	0.3047	0.03753
			2	rs898456	*CD8B2;ST6GAL2*	0.3263	0.04692
			2	rs1551718	*CD8B2;ST6GAL2*	0.294	0.0262
			2	rs4487086	*CD8B2;ST6GAL2*	3.064	0.04701
			2	rs2377685	*CD8B2;ST6GAL2*	0.3263	0.04692
			2	rs5833209	*CD8B2;ST6GAL2*	0.3263	0.04692
			5	rs7707036	*THBS4*	4.142	0.03309
			11	rs996076	*CD44*	0.128	0.005033

CHR1	rsID	Gene ID	CHR2	rsID	Gene ID	OR	P
			11	rs996075	*CD44*	0.2378	0.01367
			11	rs7934770	*CD44*	0.1663	0.01468
			11	rs9971373	*CD82*	0.06846	0.03437
			15	rs80307614	*CD276*	0.124	0.02678
2	rs4676262	*CD8B2;ST6GAL2*	2	rs12712257	*CD8B2;ST6GAL2*	3.758	0.009475
			2	rs1006472	*CD8B2;ST6GAL2*	0.2725	0.008968
			2	rs7599975	*CD8B2;ST6GAL2*	3.72	0.01042
			2	rs898456	*CD8B2;ST6GAL2*	0.2829	0.0106
			2	rs1551718	*CD8B2;ST6GAL2*	0.2231	0.0018
			2	rs4487086	*CD8B2;ST6GAL2*	3.906	0.008172
			2	rs1448118	*CD8B2;ST6GAL2*	0.3296	0.01163
			2	rs10514794	*CD8B2;ST6GAL2*	3.861	0.009054
			2	rs6721068	*CD8B2;ST6GAL2*	3.748	0.009646
			2	rs2377685	*CD8B2;ST6GAL2*	0.2829	0.0106
			2	rs5833209	*CD8B2;ST6GAL2*	0.2829	0.0106
			2	rs2241992	*CD8B2;ST6GAL2*	4.002	0.007457
			2	rs1006413	*CD8B2;ST6GAL2*	0.3274	0.01195
			3	rs13091125	*CD200;BTLA*	0.3766	0.04963
			3	rs2633610	*CD200;BTLA*	0.3744	0.04728
			3	rs13090873	*CD200;BTLA*	0.3375	0.04782
			3	rs2633611	*CD200;BTLA*	0.3628	0.04058
			3	rs2705545	*CD200;BTLA*	0.3291	0.02335
			3	rs2633568	*CD200;BTLA*	0.3666	0.03855
			3	rs721361	*CD200;BTLA*	0.284	0.01635
			3	rs721363	*CD200;BTLA*	0.3118	0.0273
			4	rs3796868	*CD38*	4.731	0.02578
2	rs4381779	*CD8B2;ST6GAL2*	3	rs2633610	*CD200;BTLA*	0.152	0.011
			3	rs2633611	*CD200;BTLA*	0.1985	0.0202
			3	rs2705545	*CD200;BTLA*	0.2694	0.04813
			3	rs2633568	*CD200;BTLA*	0.223	0.02963
			3	rs721361	*CD200;BTLA*	0.2533	0.04018
			3	rs721363	*CD200;BTLA*	0.1884	0.01718
			4	rs3796868	*CD38*	6.058	0.0177
			5	rs1274868	*CD180;LINC02242*	34.98	0.03314
			11	rs2231454	*CD59*	13.6	0.008729
			11	rs1547059	*CD44*	33.49	0.009887
			11	rs2065004	*CD44*	29.08	0.01361
2	rs56018727	*CD8B2;ST6GAL2*	3	rs2633610	*CD200;BTLA*	0.2382	0.0495
			4	rs3796868	*CD38*	5.157	0.0382
			5	rs1274868	*CD180;LINC02242*	40.6	0.02706

CHR1	rsID	Gene ID	CHR2	rsID	Gene ID	OR	P
			11	rs1738548	*CD59*	0.2226	0.04996
			11	rs2231454	*CD59*	9.02	0.01826
			11	rs11038084	*CD82;TSPAN18*	13.06	0.03974
			19	rs3865444	*CD33*	0.2453	0.03636
2	rs74879177	*CD8B2;ST6GAL2*	5	rs1274868	*CD180;LINC02242*	47.75	0.02225
			11	rs2231454	*CD59*	6.376	0.04964
			11	rs1547059	*CD44*	26.45	0.02531
			11	rs2065004	*CD44*	23.51	0.03139
2	rs12712257	*CD8B2;ST6GAL2*	2	rs17220280	*CD8B2;ST6GAL2*	5.992	0.01518
			2	rs10514794	*CD8B2;ST6GAL2*	0.4939	0.04892
			2	rs2241992	*CD8B2;ST6GAL2*	0.4684	0.0398
			6	rs6605523	*THBS2;WDR27*	2.888	0.02448
			11	rs4755854	*CD82;TSPAN18*	0.371	0.007612
			11	rs11820438	*CD82;TSPAN18*	0.4734	0.04489
			11	rs12420889	*CD82;TSPAN18*	2.546	0.02497
			12	rs2253610	*CD27-AS1*	2.491	0.01028
			17	rs4788845	*CD300C*	0.4663	0.04658
			18	rs1788110	*CD226*	3.053	0.02123
2	rs1006472	*CD8B2;ST6GAL2*	4	rs3756243	*CD38*	5.696	0.01186
			6	rs6605523	*THBS2;WDR27*	0.3125	0.0254
			11	rs4755854	*CD82;TSPAN18*	2.815	0.009171
			11	rs12420889	*CD82;TSPAN18*	0.3783	0.02962
			12	rs2253610	*CD27-AS1*	0.3812	0.01212
			17	rs4788845	*CD300C*	2.533	0.03011
			18	rs1788110	*CD226*	0.336	0.02123
2	rs7599975	*CD8B2;ST6GAL2*	2	rs17220280	*CD8B2;ST6GAL2*	5.997	0.01541
			6	rs6605523	*THBS2;WDR27*	2.68	0.03729
			11	rs4755854	*CD82;TSPAN18*	0.3396	0.004777
			11	rs11820438	*CD82;TSPAN18*	0.4408	0.03191
			11	rs12420889	*CD82;TSPAN18*	2.818	0.01621
			12	rs2253610	*CD27-AS1*	2.564	0.01008
			18	rs1788110	*CD226*	2.743	0.03818
2	rs898456	*CD8B2;ST6GAL2*	4	rs3756243	*CD38*	5.509	0.01347
			6	rs6605523	*THBS2;WDR27*	0.3586	0.03753
			11	rs4755854	*CD82;TSPAN18*	2.853	0.008626
			11	rs12420889	*CD82;TSPAN18*	0.3783	0.02962
			12	rs2253610	*CD27-AS1*	0.3814	0.01246
			17	rs4788845	*CD300C*	2.356	0.0421
			18	rs1788110	*CD226*	0.355	0.02659
2	rs17220280	*CD8B2;ST6GAL2*	5	rs1274868	*CD180;LINC02242*	37.28	0.03487

CHR1	rsID	Gene ID	CHR2	rsID	Gene ID	OR	P
2	rs1551718	*CD8B2;ST6GAL2*	4	rs3756243	*CD38*	6.356	0.008016
			6	rs6605523	*THBS2;WDR27*	0.3175	0.01813
			11	rs4755854	*CD82;TSPAN18*	2.433	0.0235
			11	rs11820438	*CD82;TSPAN18*	2.186	0.04912
			12	rs2253610	*CD27-AS1*	0.2945	0.002879
			18	rs1788110	*CD226*	0.3515	0.02615
2	rs4487086	*CD8B2;ST6GAL2*	2	rs2241992	*CD8B2;ST6GAL2*	0.468	0.04097
			6	rs6605523	*THBS2;WDR27*	2.628	0.04828
			11	rs4755854	*CD82;TSPAN18*	0.3503	0.006748
			11	rs11820438	*CD82;TSPAN18*	0.4717	0.04905
			11	rs12420889	*CD82;TSPAN18*	2.446	0.03519
			12	rs2253610	*CD27-AS1*	2.651	0.00918
			17	rs4788845	*CD300C*	0.3817	0.02071
			17	rs35729709	*CD300E;RAB37*	2.444	0.04041
			18	rs1788110	*CD226*	3.489	0.01282
2	rs1448118	*CD8B2;ST6GAL2*	4	rs2531166	*CD38;FGFBP1*	2.133	0.04624
			6	rs6605523	*THBS2;WDR27*	0.3677	0.02986
			12	rs2253610	*CD27-AS1*	0.2963	0.0018
			17	rs4788845	*CD300C*	2.258	0.03822
			18	rs1788110	*CD226*	0.3289	0.01553
2	rs10514794	*CD8B2;ST6GAL2*	2	rs2241992	*CD8B2;ST6GAL2*	0.4743	0.04375
			11	rs4755854	*CD82;TSPAN18*	0.3203	0.004308
			11	rs11820438	*CD82;TSPAN18*	0.4391	0.03543
			11	rs12420889	*CD82;TSPAN18*	2.714	0.02289
			12	rs2253610	*CD27-AS1*	2.743	0.00888
			17	rs4788845	*CD300C*	0.4097	0.03155
			17	rs35729709	*CD300E;RAB37*	2.449	0.04166
			18	rs1788110	*CD226*	3.106	0.02443
2	rs6721068	*CD8B2;ST6GAL2*	2	rs2241992	*CD8B2;ST6GAL2*	0.48	0.04525
			6	rs6605523	*THBS2;WDR27*	2.967	0.02736
			6	rs1028296	*THBS2;WDR27*	3.495	0.0496
			11	rs4755854	*CD82;TSPAN18*	0.3655	0.0099
			11	rs12420889	*CD82;TSPAN18*	2.403	0.03936
			12	rs2253610	*CD27-AS1*	2.672	0.008569
			17	rs4788845	*CD300C*	0.4082	0.03354
			17	rs35729709	*CD300E;RAB37*	2.512	0.03619
			18	rs1788110	*CD226*	3.377	0.01571
2	rs2377685	*CD8B2;ST6GAL2*	4	rs3756243	*CD38*	5.509	0.01347
			6	rs6605523	*THBS2;WDR27*	0.3586	0.03753
			11	rs4755854	*CD82;TSPAN18*	2.853	0.008626

CHR1	rsID	Gene ID	CHR2	rsID	Gene ID	OR	P
			11	rs12420889	*CD82;TSPAN18*	0.3783	0.02962
			12	rs2253610	*CD27-AS1*	0.3814	0.01246
			17	rs4788845	*CD300C*	2.356	0.0421
			18	rs1788110	*CD226*	0.355	0.02659
2	rs5833209	*CD8B2;ST6GAL2*	4	rs3756243	*CD38*	5.509	0.01347
			6	rs6605523	*THBS2;WDR27*	0.3586	0.03753
			11	rs4755854	*CD82;TSPAN18*	2.853	0.008626
			11	rs12420889	*CD82;TSPAN18*	0.3783	0.02962
			12	rs2253610	*CD27-AS1*	0.3814	0.01246
			17	rs4788845	*CD300C*	2.356	0.0421
			18	rs1788110	*CD226*	0.355	0.02659
2	rs2241992	*CD8B2;ST6GAL2*	6	rs6605523	*THBS2;WDR27*	2.97	0.02968
			6	rs1028296	*THBS2;WDR27*	3.668	0.04276
			11	rs4755854	*CD82;TSPAN18*	0.3558	0.01009
			11	rs12420889	*CD82;TSPAN18*	2.577	0.03174
			12	rs2253610	*CD27-AS1*	2.652	0.01258
			17	rs4788845	*CD300C*	0.424	0.04447
			18	rs1788110	*CD226*	3.317	0.02031
2	rs1006413	*CD8B2;ST6GAL2*	6	rs6605523	*THBS2;WDR27*	0.3923	0.04541
			11	rs4755854	*CD82;TSPAN18*	2.126	0.04392
			11	rs11820438	*CD82;TSPAN18*	2.217	0.04217
			12	rs2253610	*CD27-AS1*	0.2726	0.001626
			18	rs1788110	*CD226*	0.3568	0.0262
3	rs11708711	*CD200;BTLA*	3	rs2633610	*CD200;BTLA*	0.3815	0.02335
			3	rs2633611	*CD200;BTLA*	0.3877	0.02505
			3	rs2633568	*CD200;BTLA*	0.423	0.04142
			3	rs721363	*CD200;BTLA*	0.4182	0.0363
			11	rs10836340	*CD44*	0.2663	0.03416
			11	rs713330	*CD44*	0.2663	0.03416
			11	rs9666607	*CD44*	0.2663	0.03416
			11	rs10769059	*CD82*	2.428	0.04662
			12	rs2253610	*CD27-AS1*	2.633	0.01007
3	rs13097590	*CD200;BTLA*	3	rs2633610	*CD200;BTLA*	0.3778	0.04508
			3	rs2633611	*CD200;BTLA*	0.3688	0.03999
			3	rs2705545	*CD200;BTLA*	0.3718	0.03688
			3	rs2633568	*CD200;BTLA*	0.3698	0.0357
			3	rs721361	*CD200;BTLA*	0.3962	0.04568
			12	rs2253610	*CD27-AS1*	3.025	0.01696
3	rs13091125	*CD200;BTLA*	3	rs721361	*CD200;BTLA*	0.3669	0.03357
			12	rs2253610	*CD27-AS1*	3.262	0.00784

CHR1	rsID	Gene ID	CHR2	rsID	Gene ID	OR	P
3	rs2633610	*CD200;BTLA*	11	rs996076	*CD44*	0.3712	0.04635
			11	rs10836340	*CD44*	0.3088	0.04626
			11	rs713330	*CD44*	0.3088	0.04626
			11	rs9666607	*CD44*	0.3088	0.04626
			15	rs80307614	*CD276*	6.871	0.04434
3	rs2633611	*CD200;BTLA*	15	rs80307614	*CD276*	6.674	0.04751
3	rs2705545	*CD200;BTLA*	11	rs1547059	*CD44*	0.06918	0.04219
3	rs721361	*CD200;BTLA*	11	rs1547059	*CD44*	0.06556	0.03859
			11	rs2065004	*CD44*	0.07228	0.04896
			12	rs2253610	*CD27-AS1*	2.413	0.03414
3	rs721363	*CD200;BTLA*	12	rs2253610	*CD27-AS1*	2.435	0.02966
			15	rs80307614	*CD276*	6.697	0.04767
4	rs3756243	*CD38*	6	rs9370732	*CD83;LINC01108*	0.1178	0.03155
			11	rs11820438	*CD82;TSPAN18*	3.723	0.02837
			20	rs6074037	*CD40;CDH22*	2.511	0.0303
			20	rs11696696	*CD40;CDH22*	3.017	0.0119
4	rs3796868	*CD38*	5	rs1438739	*THBS4*	0.2589	0.03973
			6	rs6605523	*THBS2;WDR27*	5.556	0.04436
			11	rs7935663	*CD82;TSPAN18*	11.08	0.04032
4	rs3733593	*CD38*	4	rs12513168	*CD38;FGFBP1*	0.4172	0.01376
			4	rs11944611	*CD38;FGFBP1*	0.4172	0.01376
			11	rs996075	*CD44*	0.4094	0.02633
			12	rs2253610	*CD27-AS1*	0.4804	0.03845
4	rs13136270	*CD38;FGFBP1*	4	rs12513168	*CD38;FGFBP1*	0.4172	0.01376
			4	rs11944611	*CD38;FGFBP1*	0.4172	0.01376
			11	rs996075	*CD44*	0.4094	0.02633
			12	rs2253610	*CD27-AS1*	0.4804	0.03845
4	rs2531166	*CD38;FGFBP1*	17	rs11653281	*CD300C*	3.37	0.008434
			19	rs12971800	*CD33;SIGLECL1*	5.011	0.01832
4	rs13103381	*CD38;FGFBP1*	17	rs11653281	*CD300C*	0.4424	0.03195
4	rs2532070	*CD38;FGFBP1*	17	rs11653281	*CD300C*	0.4424	0.03195
5	rs1438739	*THBS4*	11	rs11038084	*CD82;TSPAN18*	6.338	0.0244
			15	rs80307614	*CD276*	12.42	0.0417
5	rs7707036	*THBS4*	11	rs11038084	*CD82;TSPAN18*	4.291	0.0305
			15	rs80307614	*CD276*	11.74	0.04524
5	rs12054697	*THBS4*	20	rs11696696	*CD40;CDH22*	2.483	0.02072
6	rs9370732	*CD83;LINC01108*	17	rs35729709	*CD300E;RAB37*	0.06331	0.02027
11	rs1738548	*CD59*	11	rs10836340	*CD44*	5.494	0.009902
			11	rs713330	*CD44*	5.494	0.009902
			11	rs9666607	*CD44*	5.494	0.009902

CHR1	rsID	Gene ID	CHR2	rsID	Gene ID	OR	P
11	rs2231454	*CD59*	20	rs6032681	*CD40;CDH22*	8.152	0.02943
11	rs996076	*CD44*	17	rs35729709	*CD300E;RAB37*	0.3116	0.03936
11	rs996075	*CD44*	11	rs4755854	*CD82;TSPAN18*	2.317	0.04613
			17	rs35729709	*CD300E;RAB37*	0.2572	0.008151
11	rs7934770	*CD44*	17	rs35729709	*CD300E;RAB37*	0.2908	0.04477
11	rs10836340	*CD44*	12	rs1059501	*CD27-AS1*	0.3324	0.04569
			17	rs35729709	*CD300E;RAB37*	0.1666	0.01259
11	rs713330	*CD44*	12	rs1059501	*CD27-AS1*	0.3324	0.04569
			17	rs35729709	*CD300E;RAB37*	0.1666	0.01259
11	rs9666607	*CD44*	12	rs1059501	*CD27-AS1*	0.3324	0.04569
			17	rs35729709	*CD300E;RAB37*	0.1666	0.01259
11	rs1547059	*CD44*	11	rs10838327	*CD82;TSPAN18*	18.01	0.04416
			11	rs7935663	*CD82;TSPAN18*	18.32	0.04316
11	rs11607862	*CD44*	11	rs1892938	*CD248;RIN1*	2.385	0.04655
11	rs9971373	*CD82*	11	rs1892938	*CD248;RIN1*	8.104	0.0458
			15	rs80307614	*CD276*	0.0322	0.02132
11	rs4755854	*CD82;TSPAN18*	11	rs10838327	*CD82;TSPAN18*	4.441	0.02433
			11	rs7935663	*CD82;TSPAN18*	4.482	0.02385
11	rs10769062	*CD82;TSPAN18*	11	rs10838327	*CD82;TSPAN18*	4.95	0.01619
			11	rs7935663	*CD82;TSPAN18*	4.993	0.01589
11	rs12420889	*CD82;TSPAN18*	11	rs10838327	*CD82;TSPAN18*	0.1172	0.007583
			11	rs7935663	*CD82;TSPAN18*	0.1008	0.007142
11	rs7935663	*CD82;TSPAN18*	11	rs1892938	*CD248;RIN1*	0.2217	0.03546
11	rs1892938	*CD248;RIN1*	12	rs1059501	*CD27-AS1*	2.34	0.04627
12	rs2253610	*CD27-AS1*	18	rs1788112	*CD226*	0.3897	0.04951
			20	rs6032681	*CD40;CDH22*	0.4277	0.03726
			20	rs2425764	*CD40;CDH22*	0.2297	0.03829
12	rs1059501	*CD27-AS1*	17	rs10512596	*CD300LB*	0.2742	0.007169
			17	rs4789074	*CD300LB;CD300C*	0.3633	0.02706
			17	rs11652446	*CD300LB;CD300C*	0.2742	0.007169
			17	rs4788845	*CD300C*	0.3756	0.01862
17	rs11653281	*CD300C*	18	rs1788112	*CD226*	4.936	0.03833
17	rs35729709	*CD300E;RAB37*	20	rs2425759	*CD40;CDH22*	0.1388	0.02969
			20	rs2425764	*CD40;CDH22*	0.08567	0.04516
18	rs1788112	*CD226*	20	rs11569333	*CD40*	27.51	0.008793
			20	rs3765457	*CD40*	22.27	0.01109
			20	rs6032681	*CD40;CDH22*	0.26	0.03521
19	rs73922419	*CD70;TNFSF14*	20	rs2424515	*THBD;CD93*	0.05183	0.02089
			20	rs1998081	*THBD;CD93*	0.05183	0.02089
			20	rs7492	*CD93*	0.07959	0.02078

CHR1	rsID	Gene ID	CHR2	rsID	Gene ID	OR	P
			20	rs2749812	*CD93*	0.1127	0.03388

PLINK v1.07 *tool was used to perform* the SNP-SNP interactions. Significance of the SNPs was maintained at *P* <0.05. CHR, chromosome; OR, Odds ratio at 95% confidence interval.

Table S4. Significant haplotypes of THBS-CD family genes identified between the controls and patients of Cohort 2.

CHR	BP1	BP2	Gene ID	Haplotype	OR	P
1	27705565	27707859	*CD164L2*	rs2474287C-rs17162558C	0.487	0.0299
2	1.07E+08	1.07E+08	*CD8B2;ST6GAL2*	rs4381779G-rs56018727A-rs74879177G	0.373	0.0248
2	1.07E+08	1.07E+08	*CD8B2;ST6GAL2*	rs12712257A-rs1006472C-rs7599975A-rs898456A-rs17220280G-rs1551718G-rs4487086A-rs1448118G-rs10514794C-rs6721068A-rs2377685C-rs5833209A-rs2241992A-rs1006413G	0.573	0.0249
2	1.07E+08	1.07E+08	*CD8B2;ST6GAL2*	rs12712257G-rs1006472A-rs7599975G-rs898456G-rs17220280G-rs1551718A-rs4487086G-rs1448118A-rs10514794A-rs6721068G-rs2377685A-rs5833209G-rs2241992G-rs1006413A	1.7	0.0331
5	79288995	79292899	*THBS4*	rs1438739A-rs7707036G	0.502	0.0139
11	44649231	44657500	*CD82;TSPAN18*	rs4755854G-rs11820438G-rs10769062A-rs7113690G-rs7113954G-rs12420889G	0.533	0.0381
11	44649231	44657500	*CD82;TSPAN18*	rs4755854A-rs11820438A-rs10769062G-rs7113690A-rs7113954A-rs12420889A	1.69	0.0463
17	72523224	72536901	*CD300LB, CD300LB;CD300C, CD300C*	rs10512596G-rs4789074G-rs11652446A-rs4788845A	2.07	0.00694
17	72523224	72536901	*CD300LB, CD300LB;CD300C, CD300C*	rs10512596A-rs4789074A-rs11652446C-rs4788845G	0.52	0.0096
20	44769666	44771565	*CD40;CDH22*	rs6074037G-rs11696696A	0.51	0.00989
20	44755889	44757213	*CD40*	rs11569333G-rs3765457A	4.01	0.0171
20	23048733	23062927	*THBD;CD93*	rs2424515G-rs1998081A-rs7492A-rs2749812A	4.44	0.0228
20	44755889	44757213	*CD40*	rs11569333A-rs3765457G	0.273	0.026
20	44769666	44771565	*CD40;CDH22*	rs6074037A-rs11696696C	1.73	0.0294
20	23048733	23062927	*THBD;CD93*	rs2424515A-rs1998081G-rs7492G-rs2749812G	0.313	0.03

PLINK v1.07 *tool was used to perform* the haplotype analysis using general linear model. Significance of the SNPs was maintained at *P* <0.05. CHR, chromosome; OR, Odds ratio at 95% confidence interval.
